# Identification of the minimal cytolytic unit for streptolysin S and an expansion of the toxin family

**DOI:** 10.1186/s12866-015-0464-y

**Published:** 2015-07-24

**Authors:** Evelyn M. Molloy, Sherwood R. Casjens, Courtney L. Cox, Tucker Maxson, Nicole A. Ethridge, Gabriele Margos, Volker Fingerle, Douglas A. Mitchell

**Affiliations:** Carl R. Woese Institute for Genomic Biology, University of Illinois at Urbana-Champaign, Urbana, IL 61801 USA; Division of Microbiology and Immunology, Department of Pathology, University of Utah Medical School, Salt Lake City, UT 84112 USA; Department of Microbiology, University of Illinois at Urbana-Champaign, Urbana, IL 61801 USA; Department of Chemistry, University of Illinois at Urbana-Champaign, Urbana, IL 61801 USA; School of Molecular and Cellular Biology, University of Illinois at Urbana-Champaign, Urbana, IL 61801 USA; Bavarian Health and Food Safety Authority, National Reference Centre for Borrelia, Oberschleissheim, Germany

**Keywords:** Streptolysin S, *Streptococcus pyogenes*, Group A *Streptococcus*, Thiazole/oxazole-modified microcin, Cytolysin, *Borrelia burgdorferi* sensu lato, Lyme disease, Linear azole-containing peptide

## Abstract

**Background:**

Streptolysin S (SLS) is a cytolytic virulence factor produced by the human pathogen *Streptococcus pyogenes* and other *Streptococcus* species. Related “SLS-like” toxins have been characterized in select strains of *Clostridium* and *Listeria*, with homologous clusters bioinformatically identified in a variety of other species. SLS is a member of the thiazole/oxazole-modified microcin (TOMM) family of natural products. The structure of SLS has yet to be deciphered and many questions remain regarding its structure-activity relationships.

**Results:**

In this work, we assessed the hemolytic activity of a series of C-terminally truncated SLS peptides expressed in SLS-deficient *S. pyogenes*. Our data indicate that while the N-terminal poly-heterocyclizable (NPH) region of SLS substantially contributes to its bioactivity, the variable C-terminal region of the toxin is largely dispensable. Through genome mining we identified additional SLS-like clusters in diverse *Firmicutes*, *Spirochaetes* and *Actinobacteria*. Among the *Spirochaete* clusters, naturally truncated SLS-like precursors were found in the genomes of three Lyme disease-causing *Borrelia burgdorferi* sensu lato (*Bbsl*) strains. Although unable to restore hemolysis in SLS-deficient *S. pyogenes*, a *Bbsl* SLS-like precursor peptide was converted to a cytolysin using purified SLS biosynthetic enzymes. A PCR-based screen demonstrated that SLS-like clusters are substantially more prevalent in *Bbsl* than inferred from publicly available genome sequences.

**Conclusions:**

The mutagenesis data described herein indicate that the minimal cytolytic unit of SLS encompasses the NPH region of the core peptide. Interestingly, this region is found in all characterized TOMM cytolysins, as well as the novel putative TOMM cytolysins we discovered. We propose that this conserved region represents the defining feature of the SLS-like TOMM family. We demonstrate the cytolytic potential of a *Bbsl* SLS-like precursor peptide, which has a core region of similar length to the SLS minimal cytolytic unit, when modified with purified SLS biosynthetic enzymes. As such, we speculate that some *Borrelia* have the potential to produce a TOMM cytolysin, although the biological significance of this finding remains to be determined. In addition to providing new insight into the structure-activity relationships of SLS, this study greatly expands the cytolysin group of TOMMs.

**Electronic supplementary material:**

The online version of this article (doi:10.1186/s12866-015-0464-y) contains supplementary material, which is available to authorized users.

## Background

**Fig. 1:**
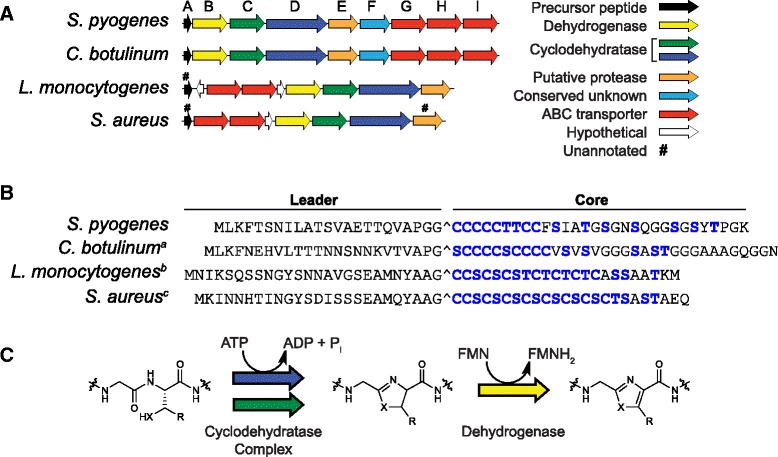
Gene cluster organization and precursor peptide sequences of SLS-like cytolysins, and post-translational modification scheme. **a** Lettering corresponds to the SLS operon “*sag*” genes. The proposed function of each gene is color-coded according to the legend. A representative is shown, as in some cases, organization varies among strains and/or not all strains of a species possess the cluster. **b** Heterocyclizable residues (Cys, Ser, Thr) are shown in blue. The predicted leader cleavage sites are indicated with carets. ^a^bioactivity of “clostridiolysin S” shown from *C. sporogenes* [17] and upon heterologous expression in *S. pyogenes ΔsagA* [7] ^b^bioactivity of “listeriolysin S” shown from native producers [19, 18], but neither the precursor peptide nor core peptide fused to SagA leader were cytolytic upon heterologous expression in *S. pyogenes ΔsagA* [7]. ^c^bioactivity of putative cytolysin not yet shown from a native producer, but core peptide fused to SagA leader cytolytic upon heterologous expression in *S. pyogenes ΔsagA* [7]. **c** Azole heterocycles are installed on a TOMM precursor peptide by the successive action of a cyclodehydratase (C and D proteins) and dehydrogenase (B protein). X = S or O; R = H or CH_3_

*Streptococcus pyogenes* (Group A *Streptococcus*) commonly causes mild diseases such as pharyngitis and impetigo, and in rare cases, invasive infections such as necrotizing fasciitis and streptococcal toxic shock syndrome [1]. While the characteristic β-hemolytic phenotype of *S. pyogenes* grown on blood agar has long been known [2], more than four decades passed before the responsible hemolytic/cytolytic factor was identified [3], streptolysin S (SLS) [4]. The eventual discovery of the SLS biosynthetic gene cluster [5] prompted investigations that subsequently designated SLS a member of the thiazole/oxazole-modified microcin (TOMM) group of natural products [6–8]. TOMMs are a class of functionally and structurally diverse ribosomal peptides that are posttranslationally modified to contain the eponymous thiazole and (methyl)oxazole heterocycles derived from select cysteine, serine, and threonine residues [8]. The SLS biosynthetic operon (Fig. 1a) encodes a precursor peptide, SagA (Fig. 1b), and three heterocycle-forming proteins, SagBCD [5, 7]. Similar to other characterized ribosomal natural products, the SagA N-terminal leader peptide contains residues recognized by the modifying enzymes, while the C-terminal core peptide undergoes heterocyclization [7, 9, 10] (Fig. 1b and c). The suspected protease, SagE, is believed to remove the leader peptide [11, 12], permitting the export of mature and bioactive SLS via a dedicated ABC transporter, SagGHI (Fig. 1a).

SLS is the founding member of a group of homologous TOMM cytolysins that have been characterized in other *Streptococcus* species (Additional file 1: Figure S1A), including invasive human isolates of the β-hemolytic Group C and Group G streptococci, which belong to *S. dysgalactiae* subsp. *equisimilis* [13]. SLS variants are also produced by the animal pathogens *S. iniae* [14] and *S. equi* [15]. More recently, *S. anginosus*, a human commensal organism and emerging opportunistic pathogen, has been shown to produce twin SLS homologs [16]. Notably, SLS-like toxins are not limited to *Streptococcus*, being produced by other *Firmicutes* including select strains of *Clostridium* [17] and *Listeria* [18–20] (Fig. 1a and b).

**Fig. 2:**
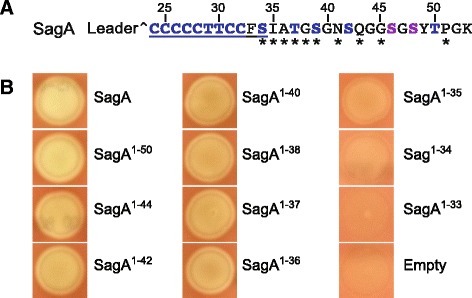
Assessment of the hemolytic activity of truncated SLS precursor peptides expressed in *S. pyogenes ΔsagA*. **a** Residues of the SagA core peptide are numbered. Potentially modified residues of SLS are blue, while those known to be heterocyclized when SagA is modified with purified SagBCD are purple. The predicted leader cleavage site is indicated with a caret. The positions of stop codons introduced in SagA are marked with asterisks (yields truncation mutants listed in panel B). The minimal core region required for hemolytic activity of SLS in *S. pyogenes ΔsagA* (SagA^24–34^) is underlined (see panel B). **b** The labels correspond to the SagA truncation mutants described in panel A. A representative colony on blood agar of GAS M1 *ΔsagA* expressing each precursor from pDCerm is shown. The colony labeled “Empty” was a negative control containing pDCerm vector

Despite intense study, the precise mechanism of SLS toxicity towards mammalian cells remains incompletely understood [21]. SLS has also thus far been recalcitrant to structural elucidation owing to its poor physicochemical properties, although an extensive mass spectral analysis detected two oxazole moieties at positions S46 and S48 of SLS modified with purified biosynthetic enzymes [17] (Fig. 2a). Previous mutagenesis studies indicate that residues spanning the entire SLS core peptide are required for *S. pyogenes* hemolysis [7, 22] (Additional file 1: Figure S1B). This contrasts with the highly conserved N-terminal poly-heterocyclizable (NPH) region and variable C-terminus of SLS natural variants (Additional file 1: Figure S1A). Here, we sought to reconcile these discrepancies by probing the contribution of the C-terminus to SLS bioactivity. We demonstrate that severely C-terminally truncated SLS peptides expressed in a *sagA* deletion mutant of *S. pyogenes* M1 5448 (*S. pyogenes ΔsagA*) retain hemolytic activity and posit that the NPH region found in all TOMM cytolysins is the defining feature of the family (Fig. 2a, underlined portion). Using bioinformatics, we identified a set of SagA homologs containing the NPH region in TOMM biosynthetic clusters in additional *Firmicutes*, as well as the *Actinobacteria* and *Spirochaete* phyla. We demonstrate that a naturally truncated precursor peptide encoded by a member of *Borrelia burgdorferi* sensu lato (*Bbsl*) species group can be matured into a cytolysin using purified SLS biosynthetic enzymes. Notably, several *Bbsl* cause Lyme disease, the most commonly reported tick-borne illness in the Northern hemisphere [23, 24], and our PCR-based screen revealed that genes encoding SLS-like precursors are prevalent in diverse *Bbsl* isolates. While our preliminary findings are intriguing, it remains to be seen if these putative cytolytic TOMMs are produced by *Borrelia*, and if so, whether they function to enhance virulence. Together, our results identify the minimal cytoytic determinant of SLS and indicate that there are potentially considerably more producers of related cytolysins than currently appreciated.

## Results and discussion

### Expression of truncated SLS precursors in *S. pyogenes ∆sagA* reveals the importance of N-terminal heterocyclizable region for hemolytic activity

An alignment of experimentally validated SLS core peptides revealed a highly conserved N-terminal region, containing 9–10 contiguous heterocyclizable residues, while the C-terminus is considerably more variable in terms of both identity of residues and length (Additional file 1: Figure S1A). Since only the NPH region of the core peptide is apparently under strict evolutionary pressure, we postulated that the C-terminal region might be less important for SLS function. Indeed, heterologous expression data support this, as peptides with divergent C-termini were able to restore the β-hemolytic phenotype in *S. pyogenes ∆sagA* [7] (Fig. 1b). To test our hypothesis, we installed premature stop codons in *sagA* (Fig. 2a) and expressed the resultant C-terminally truncated SLS derivatives in *S. pyogenes ΔsagA*. Unfortunately, the ring-state of processed SagA cannot be readily determined, even with advanced spectroscopic methods [17, 6]. We thus relied on a simple phenotypic assay, i.e. β-hemolysis on blood agar, to simultaneously evaluate successful production and export of SLS derivatives.

Consistent with the divergence of the core peptides (Additional file 1: Figure S1A), SLS retained significant activity upon C-terminal truncation. In fact, *S. pyogenes ΔsagA* expressing SLS derivatives missing up to 17 residues (SagA^1–36^) showed comparable levels of β-hemolysis on blood agar to that complimented with full-length SagA (Fig. 2b). Hemolytic activity substantially decreased with additional truncation to yield SagA^1–35^ and SagA^1–34^, with activity being undetectable in the case of SagA^1–33^ (Fig. 2b). These findings allowed us to define SagA^24–34^ as the minimal unit required for hemolytic activity of SLS expressed by *S. pyogenes*, assuming leader cleavage at the predicted site (Fig. 2a). Interestingly, this segment encompasses the entirety of the conserved NPH region (Additional file 1: Figure S1A), as well as just two additional residues (Fig. 2a). Based on the predicted leader peptide cleavage site [22], this indicates that only 11 core residues are required for detectable bioactivity of SLS expressed by *S. pyogenes*.

The retention of activity by such drastically C-terminally truncated SLS derivatives expressed in *S. pyogenes ΔsagA* stands in contrast to alanine substitution studies that implicated several of the “missing” residues in SLS activity by both expression in *S. pyogenes ΔsagA* and by modification using purified enzymes (Additional file 1: Figure S1B) [7, 22]. While a K53A mutation eliminated SLS hemolytic activity from *S. pyogenes* [22], our truncation series demonstrated that its removal is not detrimental to activity (Fig. 2b). Our results are consistent with the existence of natural SLS variants that lack a terminal lysine (Additional file 1: Figure S1A). Moreover, known SLS-like toxins encoded by non-*Streptococcus* species also lack terminal lysines (Fig. 1b). Even more striking is our observation that peptides that lack S39 retain cytolytic activity (Fig. 2b). S39 was identified as a critical residue because *S. pyogenes ΔsagA* expressing SagA-S39A was devoid of cytolytic activity and avirulent in a mouse infection model [7] (Additional file 1: Figure S1B). As before, this position is not conserved in the SLS family (Additional file 1: Figure S1A) or in SLS-like toxins encoded by non-*Streptococcus* species (Fig. 1b). Finally, many of our mutants that lack the unconserved residues S46 and S48 are hemolytic to levels indistinguishable from WT. Previous mutagenesis suggested that these positions contribute to the hemolytic properties of SLS both in *S. pyogenes* and when modified with purified enzymes [7]. S46 and S48 have also been shown to be cyclized when SLS is modified with its cognate purified biosynthetic enzymes [17], although it is not known if this holds true for native SLS. Thus, many positions that were previously implicated in SLS activity by point mutation are not conserved across the SLS family and were herein found to be dispensable. In the absence of a complete structure for SLS or detailed mechanism of action, it is unclear why mutation of a residue can be more detrimental than removal, but it is possible that the “alanine-substituted” toxins adopt an unproductive topology that is incapable of lysing cells.

### Bioinformatics-guided discovery of novel TOMM cytolysins

**Fig. 3:**
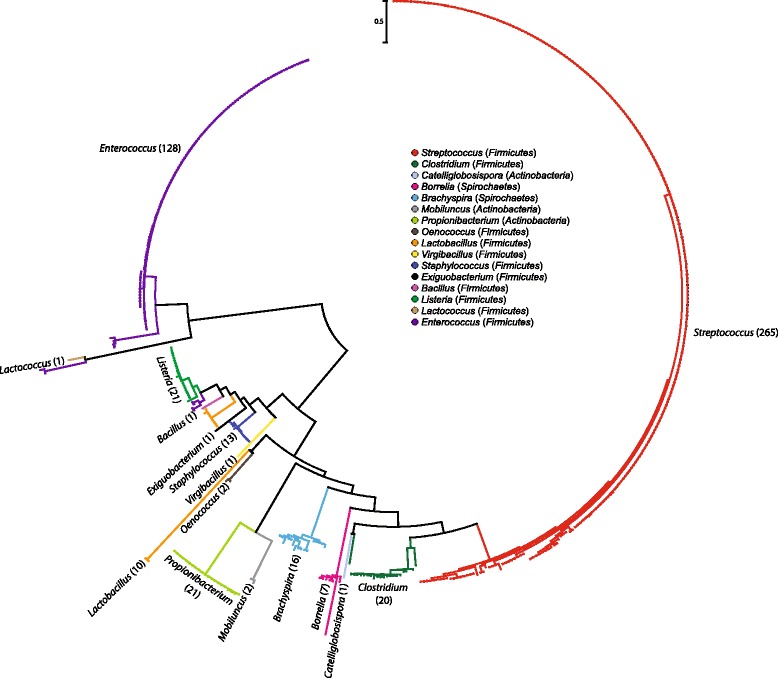
Phylogenetic analysis of the TOMM cytolysin family. A maximum-likelihood tree based on the D protein (listed in Additional file 4: Table S2) for the majority of known and putative producers of cytolytic TOMMs (as of December 2014). This phylogenetic data is deposited in the Dryad Digital Repository, doi:10.5061/dryad.d4863. Clades are color-coded based on the originating genus for each SLS-like toxin, as represented in the legend. The relevant phyla are given in parentheses

Given that the number of bioinformatically identifiable TOMM biosynthetic clusters more than quadrupled since 2011 [8, 25], we sought to determine if naturally truncated precursor peptides are present in any novel SLS-like clusters. Our genome mining effort revealed TOMM clusters encoding SagB, −C and –D homologs in the vicinity of SagA homologs in *Firmicutes* genera not previously known to possess such clusters (i.e. *Bacillus*, *Lactobacillus*, *Enterococcus*, *Exiguobacterium*, *Oenococcus* and *Virgibacillus*) (Additional file 2: Figure S2A and Additional file 3: Table S1). Interestingly, a number of *Actinobacteria* (*Mobiluncus* and *Propionibacterium*) and *Spirochaetes* (*Borrelia* and *Brachyspira*) encode SLS-like clusters (Additional file 2: Figure S2A and Additional file 3: Table S1)*.* The *Brachyspira* TOMM clusters were independently noted in a recent publication [26]. We hypothesized that the newly-annotated SLS-like precursors (Additional file 2: Figure S2B) represent novel TOMM cytolysins since the sequence of a TOMM precursor dictates the function of the natural product after posttranslational modification [8, 6]. Moreover, while the novel SagA-like precursor peptides were of variable length, all contained the aforementioned NPH region. Our identification of these putative SLS-like cytolysins greatly expands the TOMM cytolysin family (Fig. 3 and Additional file 4: Table S2) and suggests that the TOMM cytolysins are not confined to *Firmicutes*.

**Fig. 4:**
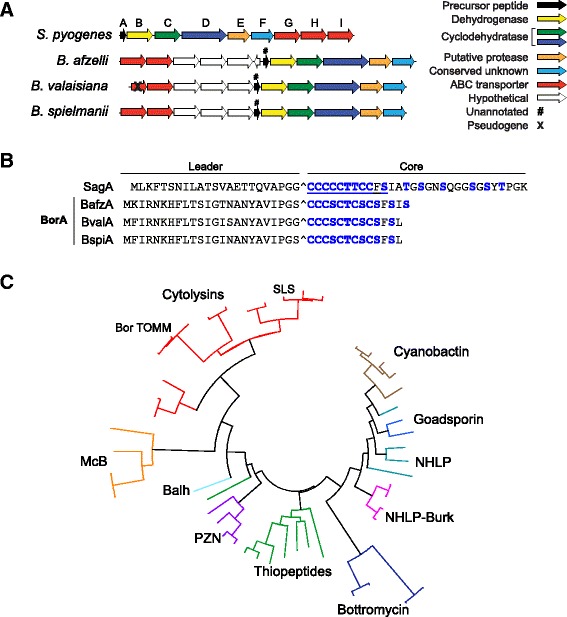
Gene cluster organization and precursor peptide sequences of SLS and Bor TOMM, and phylogenetic analysis of the TOMM family. **a** Lettering corresponds to the SLS operon “*sag*” genes. The proposed function of each gene is color-coded according to the legend. **b** BorA refers collectively to BafzA from *B. afzelii* PKo, BvalA from *B. valaisiana* VS116, and BspiA from *B. spielmanii* A14S. Potentially modified residues are shown in blue. The predicted leader cleavage sites are indicated with a caret. The minimal core region required for hemolytic activity of SLS in *S. pyogenes ΔsagA* (SagA^24–34^) is underlined. **c** A maximum-likelihood tree of a representative sample of TOMMs based on the D protein from each cluster. This phylogenetic data is deposited in the Dryad Digital Repository, doi:10.5061/dryad.d4863. Clades are color-coded based on the predicted class of TOMM natural product. NHLP, nitrile hydratase leader peptide; NHLP-Burk, NHLP from *Burkholderia*; PZN, plantazolicin; Balh, uncharacterized TOMM from *Bacillus* sp. Al Hakam; McB, microcin B from *Gammaproteobacteria*; SLS, streptolysin S; Bor TOMM, putative SLS-like cytolysin from *Bbsl*

Of particular interest were the three potential SLS-like TOMM biosynthetic clusters we discovered among the 23 published *Bbsl* genomes (Fig. 4a; hereafter referred to as “Bor TOMM clusters”), namely in *B. afzelii* PKo [GenBank: CP002947] [27], *B. valaisiana* VS116 [GenBank: CP001442] [28] and *B. spielmanii* A14S [GenBank: CP001465] [28]. The novel *Bbsl*-encoded precursor peptides were SagA homologs with core regions of similar length to our “minimal cytolytic unit” for SLS (Fig. 4b). We preliminarily classified the putative *Bbsl* SLS-like products as TOMM cytolysins based on sequence similarity to SLS, with support from a maximum likelihood tree of a subset of the TOMM family based on the associated D proteins (Fig. 4c). Thus, we assigned names to the homologous genes within the clusters following the SLS nomenclature, with *borA* encoding the precursor peptide (refers collectively to *bafzA* from *B. afzelii* PKo, *bvalA* from *B. valaisiana* VS116, and *bspiA* from *B. spielmanii* A14S; Fig. 4b), *borB* encoding the dehydrogenase and *borC/D* encoding the cyclodehydratase and so forth.

### SLS-like TOMM biosynthetic genes are widely distributed among *Bbsl*

We noted that the Bor TOMM clusters identified in *B. afzelii* PKo, *B. valaisiana* VS116 and *B. spielmanii* A14S were present on lp28-8 plasmids (Additional file 5: Figure S3). These plasmids exhibit 7-9 % nucleotide sequence divergence from each other, which is consistent with that observed on the chromosome and other linear plasmids in these species [29]. Moreover, the G + C content of the cluster is 25-26 % in all three species, which is typical for *Bbsl* plasmids. It thus appears that the Bor TOMM cluster did not arrive into the genus recently by horizontal transfer, but has likely been present for a long time, diverging in parallel with the chromosome approximately when the three species diverged.

We hypothesized that the three Bor TOMM clusters identified in *Bbsl* genomes are an underestimation of the true prevalence given that *Bbsl* are prone to losing plasmids when cultured *in vitro* [30, 31]. In order to estimate the distribution of Bor TOMM clusters in *Bbsl*, we performed a PCR screen for the associated biosynthetic genes in 140 genomic DNA samples from tick or human patient isolates (Additional file 6: Table S3). Seven *Bbsl* species were represented, originating from North America and 13 countries in Europe and Asia. Because of the similarity among the VS116, PKo, and A14S Bor TOMM cluster sequences, primers specific to *bvalB, -C* and *-D* were used to detect *borB, -C* and *-D* in the isolates (Additional file 7: Figure S4 and Additional file 8: Table S4).

Our results reveal that Bor TOMM biosynthetic genes are present in select *B. afzelii*, *B. spielmanii, B. valaisiana*, *B. lusitaniae*, and *B. garinii* isolates (Additional file 6: Table S3, summarized in Table 1). In line with the well-known plasmid instability of *Borrelia* plasmids, we observed cases of passage-dependent amplification of Bor TOMM genes (Additional file 6: Table S3). While all three biosynthetic genes could be amplified from some of the aforementioned strains, there were many instances where only one or two biosynthetic genes were detected (Table 1 and Additional file 6: Table S3). Overall, *borC* was successfully amplified more frequently than *borB* or *borD*, likely due to higher conservation at the extreme 5′ and 3′ ends where the primers were designed to anneal (Additional file 7: Figure S4). Since mutational inactivation of nonessential genes is quite common on *Bbsl* linear plasmids, another possibility is that some target genes are too divergent to detect with the primers employed [32].

**Table 1:** The Bor TOMM biosynthetic cluster is present among diverse *Bbsl* isolates

		PCR Positive			
*Bbsl* species	No. of strains screened	*borB*	*borC*	*borD*	All
*Bbss*	24	0	0	0	0
*B. garinii*	26	0	5	0	0
*B. afzelii*	47	26	34	19	14
*B. spielmanii*	7	5	5	5	5
*B. bavariensis*	13	0	0	0	0
*B. valaisiana*	21	3	15	3	3
*B. lusitaniae*	2	0	2	2	0

Positive PCR hits for Bor TOMM biosynthetic genes in *Bbsl* isolates. Strains originated in Europe and Asia, except 11 *Borrelia burgdorferi* sensu stricto (*Bbss*) from North America. “All” indicates the number of isolates testing positive for genes encoding all three putative heterocycle forming enzymes, BorBCD. More details are provided in Additional file 6: Table S3

In contrast, Bor TOMM genes were absent from all *B. burgdorferi* sensu stricto (*Bbss*) and *B. bavariensis* isolates screened (Table 1 and Additional file 6: Table S3), suggesting that they do not possess a Bor TOMM cluster, or again, that the target genes are too divergent to detect. The fact that the Bor TOMM is not present in the 13 *Bbss* published genomes [33] suggests that the former is likely true for this species. Notably, *Bbss* has been reported to produce a number of hemolytic factors [34, 35].

Mapping the distribution of a subset of the Bor TOMM cluster data onto a maximum likelihood tree, which was generated from a multilocus sequence typing (MLST) analysis, further supports our hypothesis that the Bor TOMM cluster has long been present in *Borrelia* (Additional file 9: Figure S5). The combined effects of a non-exhaustive PCR screen with non-degenerate primers and the unavoidable plasmid loss upon cultivation means that data reported here represent the minimum distribution and prevalence of the Bor TOMM cluster in *Bbsl*. However, these data demonstrate that the newly-identified Bor TOMM cluster is underrepresented in available genome sequences.

### The SLS-like peptides from *Borrelia* are potential cytolysins

**Fig. 5:**
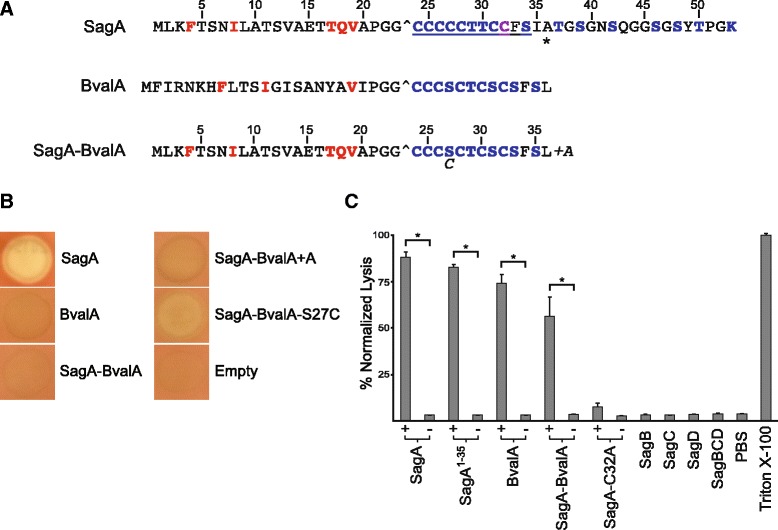
Hemolytic activity of SLS-like peptides expressed in *S. pyogenes ΔsagA* and modified by purified SagBCD enzymes. **a** Residues of the SagA and SagA-BvalA precursors are numbered. The motifs known to be important for binding SagC [7] are shown in red for the SagA leader. Partial binding motifs are also shown in red for the BvalA leader. Potentially modified residues are blue except C32, the position of a previously generated alanine substitution that abolished cytolytic activity of SagA, which is marked in purple. The predicted leader cleavage sites are indicated with a caret. The minimal core region required for hemolytic activity of SLS in *S. pyogenes ΔsagA*(SagA^24–34^) is underlined. The position of a stop codon introduced in SagA to yield the SagA^1–35^ (BvalA mimic) is marked with an asterisk. SagA-BvalA-S27C and SagA-BvalA + A point mutants are represented by italicized letters adjacent to the SagA-BvalA precursor sequence. The precursor peptide labels continue in panel B & C. **b** A representative colony on blood agar of *S. pyogenes ΔsagA* expressing each precursor peptide from pDCerm is shown. The colony labeled “Empty” was a negative control containing pDCerm vector. **c** Where appropriate, bars are labeled with + or – indicating presence or absence, respectively, of MBP-SagBCD in the reaction using the indicated MBP-tagged precursor peptide. Triton X-100, positive control for 100 % lysis; PBS, negative control for background hemolysis. Error is reported as the standard deviation of the mean (n = 3). * indicates P < 0.01 for each synthetase reaction versus the relevant unreacted precursor. P < 0.01 for all lytic samples versus all negative controls, i.e. SagA-C32A reaction, enzymes alone (labeled SagB, SagC, SagD, and SagBCD), PBS

Given that cytolytic TOMM production has been implicated in the virulence of some pathogens [7, 22, 18], coupled with the prevalence of SLS-like TOMM clusters in *Bbsl*, we decided to study the cytolytic potential of one of the naturally C-terminally truncated SLS-like peptides from *Bbsl*. In light of the complexity of *Borrelia* cultivation requirements, the aforementioned tendency to lose plasmids when cultured and inefficient genetic tools [36, 37], we opted to take a reductionist approach and assess the cytolytic potential of BvalA by heterologous expression in *S. pyogenes ΔsagA.* Importantly, there is precedent for this approach, e.g. the SLS-like precursor from *C.botulinum* (Fig. 1b) successfully complemented *S. pyogenes ΔsagA* [7] and was later confirmed to function as a cytolysin in the context of a native producer [17]. BvalA bears ~70 % amino acid similarity to SagA (Fig. 5a) and the SagBCD proteins are ~50 % similar to their BvalBCD equivalents. However, no hemolytic activity was observed upon heterologous expression of the non-cognate substrate BvalA in *S. pyogenes ΔsagA* (Fig. 5b).

Substrate binding during TOMM biosynthesis occurs via recognition of key motifs within N-terminal leader region of the precursor peptide by the C-protein component of the synthetase [25, 7]. Although partial binding motifs appear to be present in the BorA leader peptides (Fig. 5a), we cannot rule out the possibility that BvalA was not bound by the *S. pyogenes* biosynthetic machinery during heterologous expression. In light of this, we generated a chimeric construct encoding the cognate SagA leader peptide fused to the non-cognate BvalA core peptide (SagA-BvalA; Fig. 5a). Such a strategy has been exploited to replace highly divergent leader peptides with the SagA leader to allow native interaction with the heterocycle-forming synthetase [7]. In this way, hemolytic activity was demonstrated for the SLS-like core from *S. aureus* (Fig. 1b) upon expression in *S. pyogenes ΔsagA* [7]. However, SagA-BvalA was not hemolytic under identical conditions (Fig. 5b). Co-expression of rare streptococcal tRNAs [38] also failed to yield hemolysis from BvalA or SagA-BvalA, suggesting that their inactivity was not due to codon-usage bias during heterologous expression.

Returning to the native SagA precursor peptide, we noted a dramatic decrease in activity for SagA^1–35^ (which could be considered a BvalA mimic; Fig. 5a), compared to SagA^1–36^ (Fig. 2b). In case the hemolytic activity of SagA-BvalA was just below the limit of detection of this assay, we appended an alanine to the C-terminus of SagA-BvalA (SagA-BvalA + A is akin to SagA^1–36^); however, hemolysis was again not observed (Fig. 5b). While the SagA core begins with a stretch of five contiguous cysteine residues, there is a serine residue at the fourth position of the comparable region of BvalA (Fig. 5a). We thus created SagA-BvalA-S27C, which partly rescued activity (Fig. 5b), causing hemolysis only directly beneath the colony compared to the zone of clearing observed in strains with high hemolytic activity. Synthetase reactions with reconstituted TOMM biosynthetic enzymes have established that cysteine is cyclized more rapidly than serine and threonine and several TOMM synthetases are known to be selective for cysteine [39, 40]. We speculate that replacing serine with cysteine partly ameliorates a bottleneck for SagBCD in processing an unnatural substrate.

The lack of activity from BvalA-derived substrates upon expression in *S. pyogenes ΔsagA* prompted us to assess cytolytic potential using purified SagBCD enzymes. Previously, SLS-like cytolysins with no detectable activity when expressed in *S. pyogenes ΔsagA* were converted to cytolysins by this approach [7]. Numerous factors contribute to this, but perhaps most importantly, high concentrations of reactants can be employed to increase the heterocyclization yield on non-optimal substrates. Additionally, this cell-free system obviates the need for export/proteolysis machinery to generate lytic entities [6, 7]. Attempts to obtain sufficiently pure, full-length and active recombinant BvalBCD were unsuccessful; however, after treatment with the SagBCD homologs (Additional file 3: Table S1C), BvalA and SagA-BvalA were both converted into cytolysins with activity comparable to SagA and SagA^1–35^ (Fig. 5c). Omission of either the peptide substrate or SagBCD resulted in no lysis, similar to the non-lytic C32A mutant of SagA [7]. These data indicated that the BvalA precursor peptide has the potential to be converted into a cytolysin under certain conditions.

The majority of non-cognate and mutant precursor peptides that become cytolytic upon treatment with purified SagBCD were also lytic when constitutively expressed in *S. pyogenes ΔsagA* [7]. However, there is precedent for the failure of *bona fide* SLS-like precursors to restore hemolysis in *S. pyogenes ΔsagA*, e.g. as was the case with listeriolysin S (Fig. 1b) [18, 7]. The contrasting results for BvalA and SagA-BvalA (Fig. 5b and c) are reminiscent of the chimeric *L. monocytogenes* substrate, SagA-LlsA, which was inactive in *S. pyogenes ΔsagA* but was transformed into a cytolysin by purified SagBCD [7]. These results underscore the strict requirements for efficient enzymatic processing and toxin export in *S. pyogenes*. Nevertheless, the ability of purified SagBCD to convert BvalA into a cytolysin, coupled with the retention of activity by truncated SagA peptides upon functional expression, suggests that the short SagA homologs found in *Bbsl* may function as cytolysins in the context of their native producers. However, whether this activity is the primary function of the Bor TOMM (if produced) requires additional study.

## Conclusions

The results of our SagA truncation series indicate that, of the 30 predicted to comprise the core peptide, the first 11 residues (SagA^24–34^) are sufficient for detectable hemolytic activity of SLS when expressed in *S. pyogenes ΔsagA*. Thus, our data indicates that the C-terminus of SagA is largely dispensable and points to the NPH region as central to SLS functionality, consistent with the conservation of this motif in natural SLS variants as well as the greater TOMM cytolysin family. Similar regions consisting of contiguous heterocyclizable residues exist in the newly-described SLS-like precursors encoded in multiple genera within *Firmicutes*, *Spirochaetes* and *Actinobacteria*. Although the ability of BvalA and SagA^1–35^ to be matured into cytolysins by purified SagBCD suggests that the “minimal cytolytic unit” may be a general feature of TOMM cytolysins, additional study will be required to determine if this holds true for all family members, especially those with longer stretches of heterocyclizable residues (e.g. listeriolysin S). In order to comprehend our observation that much of the hemolytic activity of SLS can be attributed to a mere 11 residues, a complete chemical structure of SLS and greater molecular insight into the interaction of SLS with the cellular membrane will be required.

Our identification of novel SLS-like biosynthetic clusters greatly expands the TOMM cytolysin family. Of particular interest is our discovery that many pathogenic *Bbsl* harbor the genetic capacity to produce naturally truncated SLS-like toxins. Although confirmation is needed, the most probable function for the SLS-like biosynthetic clusters in *Bbsl* is to generate a cytolytic factor, akin to the virulence-enhancing SLS of *S. pyogenes*. Thus, the hypothetical “borreliolysin S” toxin we identify herein warrants further investigation as it would represent, to the best of our knowledge, the first posttranslationally modified natural product from any Spirochaete.

In summary, this work provides evidence that the core NPH motif represents the defining feature of the SLS-like TOMM family, as well as providing a foundation for studies of the potential utilization of cytolytic TOMMs by the diverse organisms described herein.

## Methods

### Bacterial culture conditions

Strains and plasmids used in this study are listed in Additional file 10: Table S5, except *Bbsl* strains which are listed in Additional file 6: Table S3. *S. pyogenes* were grown at 37 °C in Todd–Hewitt broth or agar plates, or on plates of 5 % sheep blood in tryptic soy agar (Hardy Diagnostics) when determining hemolytic activity by functional expression. For antibiotic selection, 5 μg ml^−1^ erythromycin was used. *E. coli* strains were grown at 37 °C with aeration in Luria–Bertani broth or on Luria–Bertani agar plates; antibiotic selection utilized 500 μg ml^−1^ erythromycin and 50 μg ml^−1^ kanamycin where relevant.

### Genome mining for novel SLS-like clusters

The majority of TOMM clusters encode a precursor peptide adjacent to SagB, −C and -D homologs [8, 10], enabling their bioinformatic detection. Thus, novel SLS-like clusters were identified by BLAST-P of known SLS-like D proteins (SagD and homologs from *C. botulinum*, *S. aureus*, *L. monocytogenes*) as the query sequence. After identification of BvalD, BmurD, LactD, and EnduD, these sequences were used as a query sequence to find other homologs, with a BLAST e-value of 1e-30 as a cutoff. Subsequently, homologs of other *sag* genes were identified in the local genomic region of a single representative cluster from each genus. Manual searches were performed in the intergenic regions of the representative clusters for short, unannotated genes encoding recognizable TOMM precursors, which are usually overlooked by gene-identification algorithms. Because of inherent duplications in RefSeq, only a single representative sequence was used from organisms with no strain information. Genome clusters with low e-values were annotated manually for precursor peptides and genomic regions that resembled known cytolysins.

### Maximum likelihood phylogenetic analysis

The D proteins of a diverse set of putative TOMM cytolysin producers were analyzed because it is known that the B and C proteins can sometimes be omitted from TOMM biosynthetic gene clusters, whereas D proteins are always present [10]. Further, the B and C proteins are homologous to non-TOMM dehydrogenases and the E1-ubiquitin activating family of enzymes, and thus are prone to bioinformatic false-positives. Thus, a D protein alignment was created using the standard parameters of ClustalW [41] and then a maximum likelihood tree was made using the standard parameters of Molecular Evolutionary Genetics Analysis (MEGA) [42]. MLST phylogenetic analysis was generated in MEGA 5.1 using concatenated sequences of eight chromosomally-located housekeeping genes (*clpA*, *clpX*, *nifS*, *pepX*, *pyrG*, *recG*, *rplB*, and *uvrA*) [43] using the maximum likelihood method with 1,000 bootstrap repeats, the GTR model, uniform rates among sites and nearest-neighbor-interchange.

### PCR screen for Bor TOMM biosynthetic genes

Genomic DNA of cultured *Bbsl* isolates was purified using either a Qiagen DNeasy blood and tissue kit or a High Pure PCR Template Preparation Kit (Roche Diagnostics) [44, 45]. Total DNA from field-collected questing ticks was extracted using alkaline hydrolysis [46]. Genomic DNA from each *Bbsl* isolate was tested for the presence of *borB, −C,* and *-D* by *Taq* PCR with primers specific to VS116 (Additional file 8: Table S4). Amplification of a product of appropriate length as determined by agarose gel electrophoresis was considered a positive hit. Sanger sequencing confirmed the identity of PCR amplicons from each species. Primers that effectively amplified *Bbsl* 16S rRNA gene sequences were used to verify that sufficient DNA was present in all samples.

### Preparation of plasmids for *S. pyogenes* transformation

The chemically synthesized genes (Integrated DNA Technologies) encoding BvalA and SagA-BvalA were subcloned into pDCerm as previously described [7]. Stop codons were introduced by site-directed mutagenesis. Mutant candidates were miniprepped and verified by DNA sequencing using pDCerm_SeqMCS_F. Primers are listed in Additional file 8: Table S4. In order to obtain sufficient plasmid DNA for transformation of *S. pyogenes ΔsagA,* each pDCerm construct was maxiprepped using an E.Z.N.A. Plasmid Maxi Kit (Omega Bio-tek).

### *S. pyogenes* transformation

*S. pyogenes ΔsagA* [22] was rendered electrocompetent using a modified version of a previously published method [47]. Briefly, *S. pyogenes ΔsagA* was grown to an OD_600_ of 0.35 in 50 ml Todd–Hewitt broth with 0.6 % (w/v) glycine. Cells were washed three times with cold 0.625 M sucrose (pH 4) and resuspended in 300 μl of the same buffer with 20 % (v/v) glycerol. 50 μl of cells was equilibrated with ~1 μg of plasmid DNA on ice for 30 min and electroporated at 1.65 kV with a BioRad Micropulser. Immediately, cells were added to 250 μl Todd–Hewitt broth with 0.25 M sucrose and recovered for 2 h at 37 °C, before plating. The *S. pyogenes*-nature of transformants [48] and presence of pDCerm were verified by PCR (Additional file 8: Table S4).

### Reaction of SLS-like peptides with purified SagBCD

All proteins were expressed with maltose-binding protein (MBP) fused to the N-terminus. SagA, SagA-C32A [7], SagB, SagC and SagD were prepared as previously described [6]. The genes encoding BvalA and SagA-BvalA were subcloned into pET28b-MBP as previously described [6]. Site-directed mutagenesis was performed to yield pET28b-MBP-SagA^1–35^ (Additional file 8: Table S4). Precursor peptides were expressed from *E. coli* BL21(DE3)-RIPL (Stratagene) for 1 h at 30 °C after induction with 1 mM IPTG at OD_600_ of 0.6. Following standard lysis procedures, each protein was purified by amylose affinity chromatography [6]. MBP-SagB, -C and -D (10 μM each) were combined with the relevant MBP-tagged precursor peptide (40 μM) and allowed to react in synthetase buffer as previously described [6]. The products of the synthetase reactions were applied to washed erythrocytes and lysis assessed as previously described [6]. Hemoglobin absorbance was measured at 410 nm on a Tecan Infinite® 200 PRO plate reader. Values were normalized to a Triton X-100 positive control. Phosphate buffered saline (PBS) served as a negative control.

### Availability of supporting data

The data sets supporting the results of this article are available in the Dryad Digital Repository (http://datadryad.org/), doi:10.5061/dryad.d4863.

## Additional files

Additional file 1: Figure S1. Alignment of experimentally validated streptococcal SLS variants and SLS structure-activity relationships. (A) Alignment using Clustal Omega [49] reveals that the SLS core region possesses a highly conserved N-terminus containing 9–10 contiguous heterocylizable residues (underlined), while the C-terminus is variable in terms of both identity of residues and length. This phylogenetic data is deposited in the Dryad Digital Repository, doi:10.5061/dryad.d4863. The putative leader peptide cleavage sites are shown as carets. Residues of SagA from *S. pyogenes* are numbered. C-terminal residues where alanine substitutions were previously installed and that are specifically referred to in the article are marked in red (see panel B). (B) Summary of cytolytic activity of SagA core peptide mutants measured by expression in *S. pyogenes* Δ*sagA*. Activity on blood agar equal to complementation with wild-type SagA is designated as +++, 30–70 % activity is ++, less than 30 % activity is +, and non-detectable activity is –. C-terminal mutations specifically referred to in the article are marked in red. Mutants were expressed from pDCerm under control of a constitutive promoter [7] or from pAD under control of the native *sagA* promoter [22]. Additional file 2: Figure S2. Gene cluster organization and precursor peptide sequences of predicted SLS-like TOMM biosynthetic gene clusters. (A) One example is given for each genus with strain abbreviations as per Additional file 3: Table S1A. Lettering corresponds to the SLS operon “*sag*” genes. The proposed function of each gene is color-coded according to the legend. In the case of *Lactococcus* and *Catelliglobosispora* (included in Fig. 3), the protein sequences are not associated with a genome in GenBank, making it difficult to confirm all surrounding genes; thus, a representative cluster organization was not given. Those clusters identified in *Borrelia* are depicted in Fig. 4 and Additional file 5: Figure S3. (B) Potentially modified residues are shown in blue. The predicted leader cleavage sites are indicated with a caret. Additional file 3: Table S1. Accession numbers and expectation values of BCD proteins from representative SLS-like TOMM clusters. (A) Strains used for the similarity analysis and the corresponding abbreviations (continued in panels B and C). (B) GenBank accession numbers (ncbi.nlm.nih.gov) of the B, C and D proteins used for the analysis. (C) BLAST e-values for the designated proteins upon comparison with the *B. valaisiana* VS116 homolog. E-values were determined using BLAST-P with standard settings and limiting the searched organisms to include only the strains in panel A, except *B. valaisiana* VS116. Additional file 4: Table S2. List of accession identifiers for D proteins of putative TOMM cytolysin clusters. The genus, species, strain, and GenBank accession identifiers are given for all D proteins used in the phylogenetic analysis (Fig. 3), with inherent duplications from GenBank RefSeq annotations deleted. Additional file 5: Figure S3. Open reading frame (ORF) diagrams for *Borrelia* lp28-8 plasmids. Four published *Bbsl* genomes harbor plasmids in the lp28-8 compatibility group [50]. As is is typical for *Bbsl* linear plasmids [51], the lp28-8 plasmids are mosaically related; gray areas between maps denote regions of sequence similarity. Lp28-8 from *B. burgdorferi* 94a lacks the Bor TOMM biosynthetic cluster so is not further discussed. The ORFs of the Bor TOMM clusters of *B. spielmanii* A14S, *B. valaisiana* VS116 and *B. afzelii* PKo are color-coded as in Fig. 1A; white arrows are hypothetical or have functions unrelated to the Bor TOMM biosynthetic cluster. The numbers above an ORF give the Pfam designation. Names of the ORFs known from type strain *B. burgdorferi* B31 are labeled “*b31_*” and are shown below the maps. Each lp28-8 carries a truncated *vls* cassette region, which allows alteration of the antigenic properties of VlsE outer surface proteins, a key pathogenic strategy. An “X” over an ORF indicates a pseudogene. In VS116, the ABC transporter gene has a substantial deletion and may be nonfunctional, suggesting that a different transporter performs export or that VS116 does not produce the Bor TOMM. Additional file 6: Table S3. PCR screen for Bor TOMM biosynthetic genes. These results provide more detail on those given in Table 1. Strains marked in gray have published genomes. P# indicates number of passages where known. Male (M), female (F) or nymph (N) is indicated for tick isolates where known. In the case of human isolates, CSF indicates isolation from cerebrospinal fluid. Disease presentation is given where known: EM, erythema migrans; LD, Lyme disease; NB, neuroborreliosis; ACA, acrodermatitis chronica atrophicans. Source abbreviations: IP, Institute Pasteur; VF, Volker Fingerle; IS, Ira Schwartz; SC, Sherwood Casjens; RM, Richard Marconi; AS, Arno Schönberg; GM: Gabriele Margos; SB, Sven Bergstrom; PR, Patricia Rosa; UM, Ulrike Munderloh; FS, Franc Strle; AvD, Alje van Dam. ^1^LGL Bayern, Germany. **Bbsl* species group currently contains 21 confirmed and proposed species, including human pathogens, suspected pathogens and non-pathogenic species [52–54]. ^a^The reported *B. afzelii* ACA-1 genome sequence does not contain Bor TOMM genes [27]; however, we detected *borBCD* in two of three ACA-1 strains of differing passage numbers. Presumably lp28-8 was present in the original ACA-1 isolate but was lost prior to the sequencing of its genome. This hypothesis is supported by a report of the sequence of ACA-1 *vls* cassette region, also missing from the published ACA-1 genome, indicating the presence of the lp28-8 in their culture [55]. ^b^ *BorBCD* were not detected in passage 7 of a PKo strain that at passage 6 had its genome sequenced [27]. In contrast, *borBCD* were detected in another isolate of PKo at passage 10, again highlighting the well-known heterogeneous nature of *Bbsl* upon cultivation. Additional file 7: Figure S4. Nucleotide alignments of Bor TOMM biosynthetic genes. *BorB* (A), *borC* (B) and *borD* (C) from *Borrelia afzelii* PKo (Bafz), *B. spielmanii* A14S (Bspi), and *B. valaisiana* VS116 (Bval) were aligned using ClustalW. This phylogenetic data is deposited in the Dryad Digital Repository, doi:10.5061/dryad.d4863. Sites where PCR screening primers annealed are underlined. The primers used to detect *borB*/*C*/*D* (Table 1 and Additional file 6: Table S3) were based on *bvalB*/*C*/*D* from *B. valaisiana* VS116 (Additional file 8: Table S4). The number of mismatches per primer when compared to the two other sequenced examples of the gene is indicated in parentheses for each. Additional file 8: Table S4. Primers used in this study. In all cases, *bvalA/B/C/D* or BvalA/B/C/D refers to the relevant TOMM genes or proteins from *B. valaisiana* VS116. Restriction endonuclease recognition sites are underlined. Lower-case letters indicate bases targeted for mutagenesis. Additional file 9: Figure S5. Maximum likelihood tree of *Bbsl* strains used in the Bor TOMM PCR screen. The tree shows the relationships of a number of *Borrelia* strains, including a subset of the *Bbsl* strains included in our PCR screen. This phylogenetic data is deposited in the Dryad Digital Repository, doi:10.5061/dryad.d4863. A plus sign (+) next to the strain designation indicates that gene(s) from the Bor TOMM biosynthetic cluster (*borB*/*borC*/*borD*) was/were detected (Additional file 6: Table S3). WGS: whole genome sequence. Additional file 10: Table S5. Strains and plasmids used in this study. In all cases, *bvalA*/BvalA refers to the TOMM gene/peptide from *B. valaisiana* VS116. The sequences of the relevant precursor peptides are shown in Figs. 2A and 5A.

## Abbreviations

SLS: Streptolysin S
TOMM: Thiazole/oxazole-modified microcin
NPH: N-terminal poly-heterocyclizable (region)
*Bbsl*: *Borrelia burgdorferi* sensu lato
*Bbss*: *Borrelia burgdorferi* sensu stricto
MLST: Multilocus sequence typing
MEGA: Molecular Evolutionary Genetics Analysis
MBP: Maltose-binding protein
PBS: Phosphate buffered saline
ORF: Open reading frame
