# Supplementary material for: Identification of the minimal cytolytic unit for streptolysin S and an expansion of the toxin family
Source: BMC Microbiol. 2015 Jul 24;15:141. doi: 10.1186/s12866-015-0464-y (PMC4513790; doi:10.1186/s12866-015-0464-y)
Supplement: Additional file 1: Figure S1. — Alignment of experimentally validated streptococcal SLS variants and SLS structure-activity relationships. (A) Alignment using Clustal Omega [49] reveals that the SLS core region possesses a highly conserved N-terminus containing 9–10 contiguous heterocylizable residues (underlined), while the C-terminus is variable in terms of both identity of residues and length. This phylogenetic data is deposited in the Dryad Digital Repository, doi:10.5061/dryad.d4863. The putative leader peptide cleavage sites are shown as carets. Residues of SagA from S. pyogenes are numbered. C-terminal residues where alanine substitutions were previously installed and that are specifically referred to in the article are marked in red (see panel B). (B) Summary of cytolytic activity of SagA core peptide mutants measured by expression in S. pyogenes ΔsagA. Activity on blood agar equal to complementation with wild-type SagA is designated as +++, 30–70 % activity is ++, less than 30 % activity is +, and non-detectable activity is –. C-terminal mutations specifically referred to in the article are marked in red. Mutants were expressed from pDCerm under control of a constitutive promoter [7] or from pAD under control of the native sagA promoter [22]. [file 12866_2015_464_MOESM1_ESM.pdf]

A

```
                     5    10   15   20    25   30   35   40    45   50
S. pyogenes        MLKFTSNILATSVAETTQVAPGG^CCCCCTTCCFSIATGSG-NSQGGSGSYTPGK--
S. dysgalactiae    MLQFTSNILATSVAETTQVAPGG^CCCCCTTCCFSINVGGG-SAQGGSGSYTPGK--
S. iniae           MLQFTSNILATSVAETTQVAPGG^CCCCCCTCCVAVNVGSG-SAQGGSGTPAPAPK-
S. equi            MLQFASNILATSVAETTQVAPGG^CCCCCSCCCCVSASWGNTTINNNYGAAEPKA--
S. anginosus_1     MLKFSSNVLATSVADTTQVAPGG^CCCCCCTCCFSVAVGGN-A-TGGSTTGSVAPTK
S. anginosus_2     MLKLDSHIMATSVAETTQVAPGG^CCCCCCTCCFSVAVGGN-A-TGGSTNIKP----
                   **:: *.::*****:******** *****  **      .
```

B

SagA point mutant	Bioactivity	Core peptide region	Reference
C24A	–	N-terminal heterocyclizable	[7] & [22]
C25A	+	N-terminal heterocyclizable	[7]
C26A	++	N-terminal heterocyclizable	[7]
C27A	+ / –	N-terminal heterocyclizable	[7] / [22]
C28A	+++	N-terminal heterocyclizable	[7]
T29V	+++	N-terminal heterocyclizable	[7]
T30V	++	N-terminal heterocyclizable	[7]
C31A	+++	N-terminal heterocyclizable	[7]
C32A	–	N-terminal heterocyclizable	[7]
S34A	+	C-terminal variable	[7]
T37A	+++	C-terminal variable	[7]
S39A	–	C-terminal variable	[7]
S42A	+++	C-terminal variable	[7]
S46A	+	C-terminal variable	[7]
S48A	++	C-terminal variable	[7]
T50V	+++	C-terminal variable	[7]
K53A	–	C-terminal variable	[22]
