# Supplementary material for: Identification of the minimal cytolytic unit for streptolysin S and an expansion of the toxin family
Source: BMC Microbiol. 2015 Jul 24;15:141. doi: 10.1186/s12866-015-0464-y (PMC4513790; doi:10.1186/s12866-015-0464-y)
Supplement: Additional file 2: Figure S2. — Gene cluster organization and precursor peptide sequences of predicted SLS-like TOMM biosynthetic gene clusters. (A) One example is given for each genus with strain abbreviations as per Additional file 3: Table S1A. Lettering corresponds to the SLS operon “sag” genes. The proposed function of each gene is color-coded according to the legend. In the case of Lactococcus and Catelliglobosispora (included in Fig. 3), the protein sequences are not associated with a genome in GenBank, making it difficult to confirm all surrounding genes; thus, a representative cluster organization was not given. Those clusters identified in Borrelia are depicted in Fig. 4 and Additional file 5: Figure S3. (B) Potentially modified residues are shown in blue. The predicted leader cleavage sites are indicated with a caret. [file 12866_2015_464_MOESM2_ESM.pdf]

A

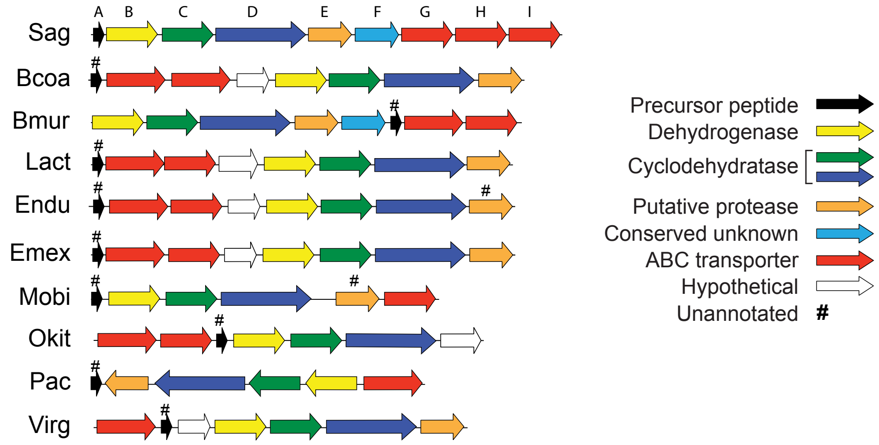

B

	Leader		Core
Sag	MLKFTSNILATSVAETTQVAPGG	^	CCCCCTTCCFSIATGSGNSQGGSGSYTPGK
Bcoa	MRIKEHRDNTYGKVTYKEAMNYAGG	^	CCSCSCSCSCSCSTCASTPARN
Bmur	MIKYSSKVRTTCSLKSQSVVLTPGG	^	CCCSCCCSCCVNVTTTTNN
Lact	MVIVEQKFNKYNHTSNMDAMEYAAG	^	CCTCTTTSCTTSCAAVAES
Endu	MNIKSQSMNGYSNTGVGAEAMNYAAG	^	CCSCSCSTCTCTCSASTATETKE
Emex	MKIKHQASSNYSEMSNGSETMHYAAG	^	SCSCSCSCSCSCSSSA
Mobi	MALATSTNVGGSPMLVTPGG	^	CTCTIGCCCSCSCGTLQTAV
Okit	MNIKKAERSFVSNNVKLNELPVAG	^	CCCCSCCFKVTINKGDE
Pac	MSSALGIGSQVDMLAPGG	^	STSSSSGSCCSCSTCAVVAQPQD
Virg	MNMKDGSIQSHSFLSTGEQTQALSYAAG	^	SCSCSCSCSCCCSSSS
