# Supplementary material for: Identification of the minimal cytolytic unit for streptolysin S and an expansion of the toxin family
Source: BMC Microbiol. 2015 Jul 24;15:141. doi: 10.1186/s12866-015-0464-y (PMC4513790; doi:10.1186/s12866-015-0464-y)
Supplement: Additional file 3: Table S1. — Accession numbers and expectation values of BCD proteins from representative SLS-like TOMM clusters. (A) Strains used for the similarity analysis and the corresponding abbreviations (continued in panels B and C). (B) GenBank accession numbers (ncbi.nlm.nih.gov) of the B, C and D proteins used for the analysis. (C) BLAST e-values for the designated proteins upon comparison with the B. valaisiana VS116 homolog. E-values were determined using BLAST-P with standard settings and limiting the searched organisms to include only the strains in panel A, except B. valaisiana VS116. [file 12866_2015_464_MOESM3_ESM.pdf]

A

Abr.	Strain
Bval	*Borrelia valasiana* VS116
Sag	*Streptococcus pyogenes* M1 GAS
Bts	*Clostridium botulinum* A str. ATCC 3502
Lls	*Listeria monocytogenes* serotype 4b str. F2365
Sts	*Staphylococcus aureus* subsp. *aureus* JKD6159
Bmur	*Brachyspira murdochii* DSM 12563
Lact	*Lactobacillus crispatus* MV-1A-US
Endu	*Enterococcus durans* ATCC 6056
Emex	*Exiguobacterium mexicanum* str. HUD
Okit	*Oenococcus kitaharae* DSM 17330
Pac	*Propionibacterium acnes* HL099PA1
Virg	*Virgibacillus alimentarius* str. J18T
Mobi	*Mobiluncus mulieris* FB024-16
Bcoa	*Bacillus coagulans* XZL9

B

	**B protein (Accession)**	**C protein (Accession)**	**D protein (Accession)**
Bval	ACN53116.1	ACN53117.1	ACN53119.1
Sag	AAK33685.1	AAK33686.1	AAK33687.1
Bts	YP_001386444.1	YP_001386445.1	YP_001386446.1
Lls	YP_013716.1	YP_013717.1	YP_013718.1
Sts	ADL23377.1	ADL23376.1	ADL23375.1
Bmur	ADG71093.1	ADG71092.1	ADG71091.1
Lact	WP_005721909.1	WP_005729239.1	WP_005721911.1
Endu	EOT25570.1	EOT25569.1	EOT25568.1
Emex	KGI86368.1	KGI86369.1	KGI86370.1
Okit	EHN58990.1	EHN58991.1	EHN58992.1
Pac	EGF73207.1	EGF73206.1	EGF73205.1
Virg	WP_029270587.1	Wp_029270585.1	WP_02970583.1
Mobi	EFN92400.1	EFN92374.1	EFN92445.1
Bcoa	WP_017550909.1	WP_026104562.1	WP_017550911.1

C

	**B protein (e-value)**	**C protein (e-value)**	**D protein (e-value)**
Bval	0	0	0
Sag	2e-37	1e-29	1e-58
Bts	2e-40	4e-51	5e-80
Lls	4e-38	3e-06	2e-36
Sts	4e-39	3e-06	2e-40
Bmur	3e-33	2e-38	9e-67
Lact	1e-34	3e-04	9e-37
Endu	8e-37	3e-07	4e-36
Emex	4e-37	2e-05	2e-32
Okit	2e-33	2e-13	1e-47
Pac	2e-23	2e-04	5e-16
Virg	2e-38	3e-08	1e-44
Mobi	1e-19	7e-10	3e-22
Bcoa	2e-36	1e-08	1e-34
