# Supplementary figures and images for: Identification of the minimal cytolytic unit for streptolysin S and an expansion of the toxin family
Source: BMC Microbiol. 2015 Jul 24;15:141. doi: 10.1186/s12866-015-0464-y (PMC4513790; doi:10.1186/s12866-015-0464-y)

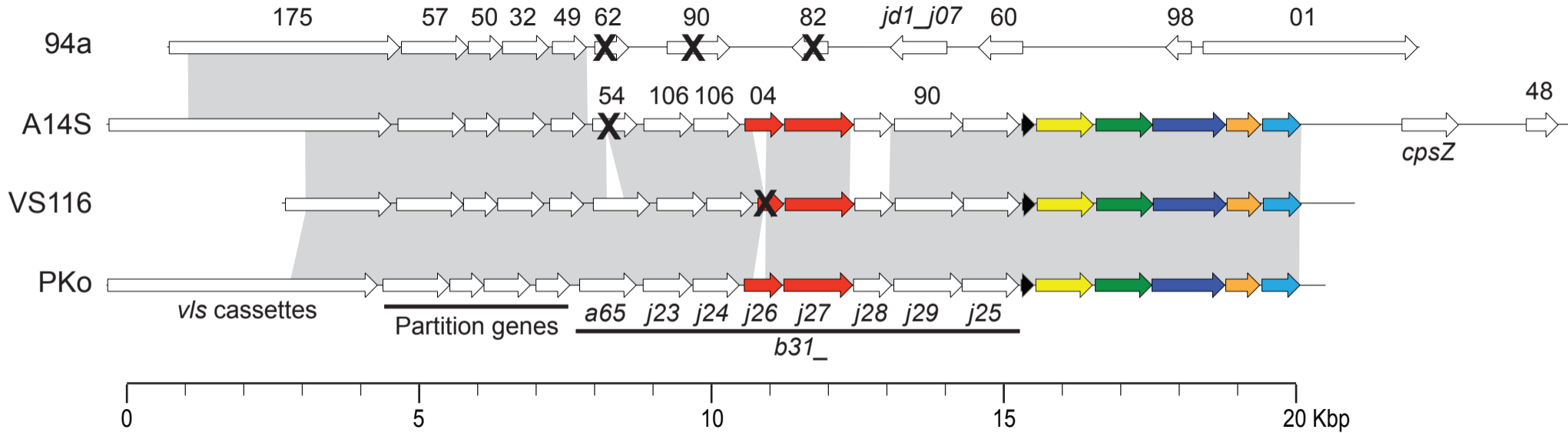

Supplement: Additional file 5: Figure S3. — Open reading frame (ORF) diagrams for Borrelia lp28-8 plasmids. Four published Bbsl genomes harbor plasmids in the lp28-8 compatibility group [50]. As is is typical for Bbsl linear plasmids [51], the lp28-8 plasmids are mosaically related; gray areas between maps denote regions of sequence similarity. Lp28-8 from B. burgdorferi 94a lacks the Bor TOMM biosynthetic cluster so is not further discussed. The ORFs of the Bor TOMM clusters of B. spielmanii A14S, B. valaisiana VS116 and B. afzelii PKo are color-coded as in Fig. 1A; white arrows are hypothetical or have functions unrelated to the Bor TOMM biosynthetic cluster. The numbers above an ORF give the Pfam designation. Names of the ORFs known from type strain B. burgdorferi B31 are labeled “b31_” and are shown below the maps. Each lp28-8 carries a truncated vls cassette region, which allows alteration of the antigenic properties of VlsE outer surface proteins, a key pathogenic strategy. An “X” over an ORF indicates a pseudogene. In VS116, the ABC transporter gene has a substantial deletion and may be nonfunctional, suggesting that a different transporter performs export or that VS116 does not produce the Bor TOMM. [file 12866_2015_464_MOESM5_ESM.pdf]

**Supplementary Figure 5**

**
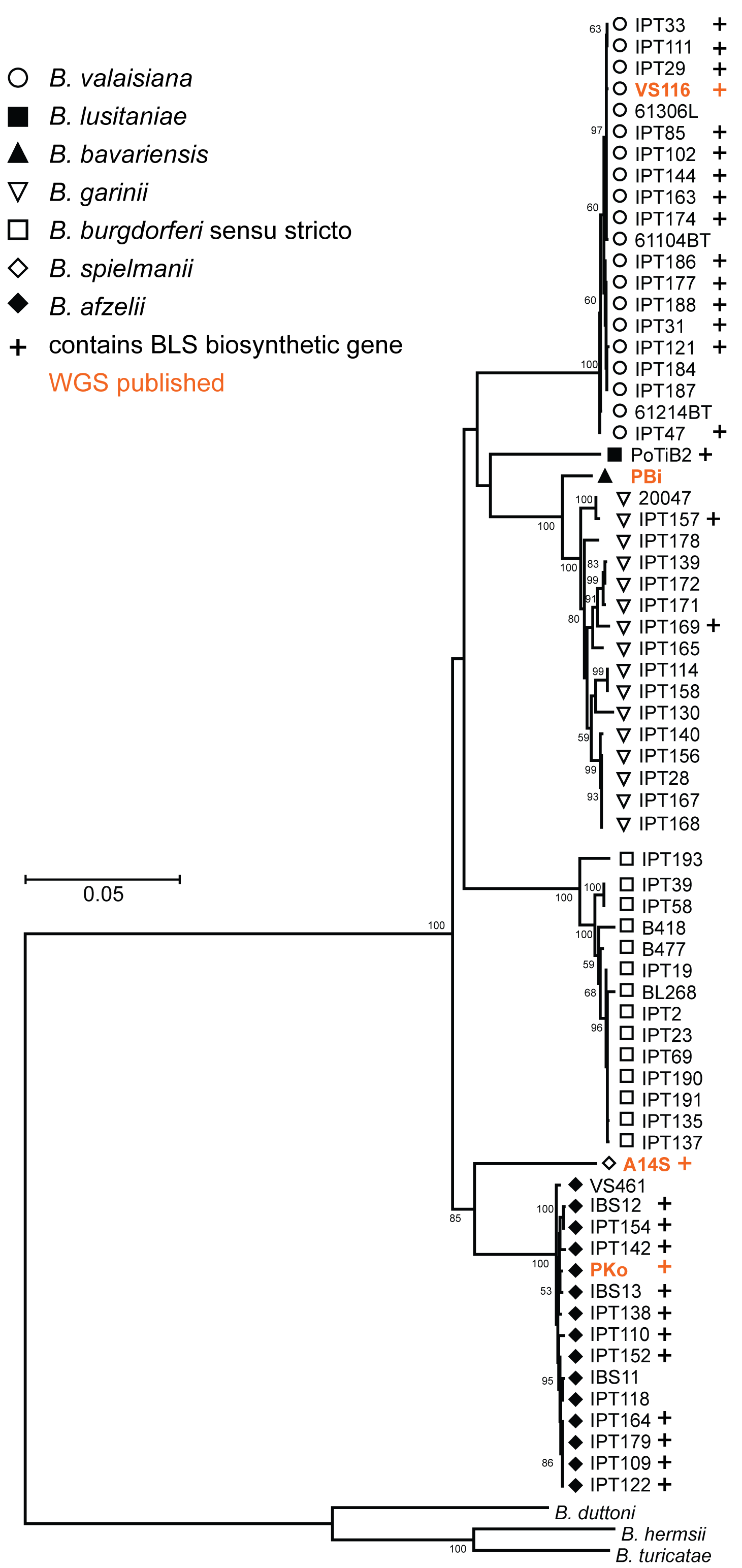
**

Supplement: Additional file 9: Figure S5. — Maximum likelihood tree of Bbsl strains used in the Bor TOMM PCR screen. The tree shows the relationships of a number of Borrelia strains, including a subset of the Bbsl strains included in our PCR screen. This phylogenetic data is deposited in the Dryad Digital Repository, doi:10.5061/dryad.d4863. A plus sign (+) next to the strain designation indicates that gene(s) from the Bor TOMM biosynthetic cluster (borB/borC/borD) was/were detected (Additional file 6: Table S3). WGS: whole genome sequence. [file 12866_2015_464_MOESM9_ESM.docx]
