# Supplementary material for: Identification of the minimal cytolytic unit for streptolysin S and an expansion of the toxin family
Source: BMC Microbiol. 2015 Jul 24;15:141. doi: 10.1186/s12866-015-0464-y (PMC4513790; doi:10.1186/s12866-015-0464-y)
Supplement: Additional file 6: Table S3. — PCR screen for Bor TOMM biosynthetic genes. These results provide more detail on those given in Table 1. Strains marked in gray have published genomes. P# indicates number of passages where known. Male (M), female (F) or nymph (N) is indicated for tick isolates where known. In the case of human isolates, CSF indicates isolation from cerebrospinal fluid. Disease presentation is given where known: EM, erythema migrans; LD, Lyme disease; NB, neuroborreliosis; ACA, acrodermatitis chronica atrophicans. Source abbreviations: IP, Institute Pasteur; VF, Volker Fingerle; IS, Ira Schwartz; SC, Sherwood Casjens; RM, Richard Marconi; AS, Arno Schönberg; GM: Gabriele Margos; SB, Sven Bergstrom; PR, Patricia Rosa; UM, Ulrike Munderloh; FS, Franc Strle; AvD, Alje van Dam. 1LGL Bayern, Germany. *Bbsl species group currently contains 21 confirmed and proposed species, including human pathogens, suspected pathogens and non-pathogenic species [52–54]. aThe reported B. afzelii ACA-1 genome sequence does not contain Bor TOMM genes [27]; however, we detected borBCD in two of three ACA-1 strains of differing passage numbers. Presumably lp28-8 was present in the original ACA-1 isolate but was lost prior to the sequencing of its genome. This hypothesis is supported by a report of the sequence of ACA-1 vls cassette region, also missing from the published ACA-1 genome, indicating the presence of the lp28-8 in their culture [55]. b BorBCD were not detected in passage 7 of a PKo strain that at passage 6 had its genome sequenced [27]. In contrast, borBCD were detected in another isolate of PKo at passage 10, again highlighting the well-known heterogeneous nature of Bbsl upon cultivation. [file 12866_2015_464_MOESM6_ESM.docx]

**Supplementary Table 3**

|  |  |  |  |  | **Presence of gene** | | | | |  |  |
| --- | --- | --- | --- | --- | --- | --- | --- | --- | --- | --- | --- |
| ***Bbsl** species** | **Strain** | **Geographic Origin** | **Biological Source** | **P#** | ***borB*** | ***borC*** | | ***borD*** | | **Source** | |
| ***B. burgdorferi* sensu stricto**  **(pathogen, North America and Western Europe)** | B297 | New York | Skin | 4 | **-** | | **-** | | **-** | IS | |
|  | B331 | New York (Westchester County) | Skin (EM) | 5 | **-** | | **-** | | **-** | IS | |
|  | B356 | New York (Westchester County) | Skin (EM) | 3 | **-** | | **-** | | **-** | IS | |
|  | B376 | New York (Westchester County) | Skin (EM) | 4 | **-** | | **-** | | **-** | IS | |
|  | B379 | New York | Skin (EM) | 5 | **-** | | **-** | | **-** | IS | |
|  | B418 | New York (Westchester County) | Skin (EM) | 2 | **-** | | **-** | | **-** | IS | |
|  | B477 | New York | Skin (EM) | 2 | **-** | | **-** | | **-** | IS | |
|  | B500 | New York (Westchester County) | Skin (EM) | 2 | **-** | | **-** | | **-** | IS | |
|  | B515 | New York (Westchester County) | Skin (EM) | 4 | **-** | | **-** | | **-** | IS | |
|  | BL206 | New York (Westchester County) | Blood (LD) | 4 | **-** | | **-** | | **-** | IS | |
|  | BL268 | New York | Blood | 3 | **-** | | **-** | | **-** | IS | |
|  | IPT2 | France (Alsace) | *I. ricinus* (M) | Unknown | **-** | | **-** | | **-** | IP | |
|  | IPT19 | France (Alsace) | *I. ricinus* (M) | Unknown | **-** | | **-** | | **-** | IP | |
|  | IPT23 | France (Alsace) | *I. ricinus* (F) | Unknown | **-** | | **-** | | **-** | IP | |
|  | IPT39 | France (Alsace) | *I. ricinus* (M) | Unknown | **-** | | **-** | | **-** | IP | |
|  | IPT58 | France (Alsace) | *I. ricinus* (F) | Unknown | **-** | | **-** | | **-** | IP | |
|  | IPT69 | France (Alsace) | *I. ricinus* (N) | Unknown | **-** | | **-** | | **-** | IP | |
|  | IPT135 | France (Auvergne) | *I. ricinus* (F) | Unknown | **-** | | **-** | | **-** | IP | |
|  | IPT137 | France (Alsace) | *I. ricinus* (F) | Unknown | **-** | | **-** | | **-** | IP | |
|  | IPT190 | France (Normandie) | *I. ricinus* (M) | Unknown | **-** | | **-** | | **-** | IP | |
|  | IPT191 | France (Normandie) | *I. ricinus* (F) | Unknown | **-** | | **-** | | **-** | IP | |
|  | IPT193 | France (Normandie) | *I. ricinus* (M) | Unknown | **-** | | **-** | | **-** | IP | |
|  | PAli | Germany (Regensburg) | Vitreous body | 1 | **-** | | **-** | | **-** | VF | |
|  | PDri | Germany (Munich) | Skin (EM & arthritis) | 2 | **-** | | **-** | | **-** | VF | |
| ***B. garnii***  **(pathogen, Eurasia)** | IPT28 | France (Alsace) | *I. ricinus* (M) | Unknown | **-** | | **-** | | **-** | IP | |
|  | IPT114 | France (Alsace) | *I. ricinus* (M) | Unknown | **-** | | **-** | | **-** | IP | |
|  | IPT130 | France (Alsace) | *I. ricinus* (M) | Unknown | **-** | | **-** | | **-** | IP | |
|  | IPT139 | France (Alsace) | *I. ricinus* (F) | Unknown | **-** | | **-** | | **-** | IP | |
|  | IPT140 | France (Alsace) | *I. ricinus* (M) | Unknown | **-** | | **-** | | **-** | IP | |
|  | IPT156 | France (Auvergne) | *I. ricinus* (M) | Unknown | **-** | | **-** | | **-** | IP | |
|  | IPT157 | France (Limousin) | *I. ricinus* (F) | Unknown | **-** | | **+** | | **-** | IP | |
|  | IPT158 | France (Limousin) | *I. ricinus* (M) | Unknown | **-** | | **-** | | **-** | IP | |
|  | IPT165 | France (Auvergne) | *I. ricinus* (M) | Unknown | **-** | | **-** | | **-** | IP | |
|  | IPT167 | France (Limousin) | *I. ricinus* (F) | Unknown | **-** | | **-** | | **-** | IP | |
|  | IPT168 | France (Limousin) | *I. ricinus* (M) | Unknown | **-** | | **-** | | **-** | IP | |
|  | IPT169 | France (Auvergne) | *I. ricinus* (F) | Unknown | **-** | | **+** | | **-** | IP | |
|  | IPT171 | France (Auvergne) | *I. ricinus* (F) | Unknown | **-** | | **-** | | **-** | IP | |
|  | IPT172 | France (Auvergne) | *I. ricinus* (F) | Unknown | **-** | | **-** | | **-** | IP | |
|  | IPT178 | France (Auvergne) | *I. ricinus* (F) | Unknown | **-** | | **-** | | **-** | IP | |
|  | PBes | Germany (Minden) | CSF (NB) | 5 | **-** | | **+** | | **-** | VF | |
|  | PBol | Germany (Nurnberg) | CSF (NB) | 3 | **-** | | **-** | | **-** | VF | |
|  | PBr | Germany (Günzburg) | CSF (NB) | 7 | **-** | | **-** | | **-** | VF | |
|  | PBu | Germany (Ulm) | Skin (EM) | 4 | **-** | | **+** | | **-** | VF | |
|  | PFr | Germany (Munich) | CSF (NB) | 6 | **-** | | **-** | | **-** | VF | |
|  | PHei | Germany (Göttingen) | CSF (NB) | 6 | **-** | | **-** | | **-** | VF | |
|  | PKi | Germany (Tübingen) | CSF (NB) | 11 | **-** | | **-** | | **-** | VF | |
|  | PLa | Germany (Koblenz) | CSF (NB) | 5 | **-** | | **-** | | **-** | VF | |
|  | PRef | Germany (Günzburg) | CSF (NB) | 11 | **-** | | **+** | | **-** | VF | |
|  | PWudII | Germany (Munich) | Skin (EM) | 5 | **-** | | **-** | | **-** | VF | |
|  | 20047 | France | *I. ricinus* | 3 | **-** | | **-** | | **-** | SC from RM | |
| ***B. afzelii***  **(pathogen, Eurasia)** | ACA-1^a^ | Sweden | Skin (ACA) | <10 | **+** | | **+** | | **+** | SC from SB | |
|  | ACA-1^a^ | Sweden | Skin (ACA) | 3 | **+** | | **+** | | **+** | SC from IS | |
|  | ACA-1^a^ | Sweden | Skin (ACA) | 4 | **-** | | **-** | | **-** | SC from IS | |
|  | B023 | Germany | Skin (EM) | <10 | **-** | | **-** | | **-** | SC from UM | |
|  | DK26 | Denmark | Skin (EM) | <10 | **-** | | **-** | | **-** | SC from RM | |
|  | EMC1 | Sweden | Skin (EM) | 3 | **-** | | **-** | | **+** | SC from RM | |
|  | HT10 | Japan | *I. persulcatus* | <10 | **+** | | **+** | | **+** | SC from RM | |
|  | IBS11 | France (Alsace) | Skin (EM) | Unknown | **-** | | **-** | | **-** | IP | |
|  | IBS12 | France (Alsace) | Skin (EM) | Unknown | **+** | | **+** | | **-** | IP | |
|  | IBS13 | France (Alsace) | Skin (ACA) | Unknown | **-** | | **+** | | **-** | IP | |
|  | IPT109 | France (Alsace) | *I. ricinus* (F) | Unknown | **-** | | **+** | | **-** | IP | |
|  | IPT110 | France (Alsace) | *I. ricinus* (F) | Unknown | **+** | | **+** | | **-** | IP | |
|  | IPT118 | France (Auvergne) | *I. ricinus* (F) | Unknown | **-** | | **-** | | **-** | IP | |
|  | IPT122 | France (Auvergne) | *I. ricinus* (F) | Unknown | **+** | | **+** | | **-** | IP | |
|  | IPT138 | France (Alsace) | *I. ricinus* (F) | Unknown | **+** | | **+** | | **-** | IP | |
|  | IPT142 | France (Alsace) | *I. ricinus* (M) | Unknown | **-** | | **+** | | **-** | IP | |
|  | IPT152 | France (Limousin) | *I. ricinus* (M) | Unknown | **-** | | **+** | | **-** | IP | |
|  | IPT154 | France (Limousin) | *I. ricinus* (F) | Unknown | **-** | | **+** | | **-** | IP | |
|  | IPT164 | France (Auvergne) | *I. ricinus* (M) | Unknown | **+** | | **+** | | **-** | IP | |
|  | IPT179 | France (Auvergne) | *I. ricinus* (M) | Unknown | **+** | | **+** | | **-** | IP | |
|  | Isau1 | Germany (Munich) | *I. ricinus* | 4 | **+** | | **+** | | **+** | VF | |
|  | J1 ("IPF") | Japan | *I. persulcatus* | <10 | **-** | | **+** | | **-** | SC from RM | |
|  | PBas | Germany (Munich) | Skin (borrelial lymphocytoma) | 2 | **+** | | **+** | | **+** | VF | |
|  | PBec | Germany (Hamburg) | Skin (ACA) | 3 | **+** | | **+** | | **-** | VF | |
|  | PBil | Germany (Minden) | Skin (EM) | 4 | **+** | | **+** | | **-** | VF | |
|  | PBo | Germany (Munich) | CSF (NB) | 3 | **+** | | **+** | | **+** | VF | |
|  | PBog | Germany (Berchtesgarden) | Skin (EM) | 6 | **+** | | **+** | | **+** | VF | |
|  | PEbe | Germany (Altötting) | Skin (EM) | 2 | **+** | | **+** | | **+** | VF | |
|  | PFiII | Germany (Munich) | Skin (ACA) | 6 | **-** | | **+** | | **-** | VF | |
|  | PFuk | Germany (Munich) | Skin (ACA) | 3 | **+** | | **+** | | **+** | VF | |
|  | PHak | Germany (Munich) | Skin (EM) | 3 | **+** | | **+** | | **+** | VF | |
|  | PHes | Germany (Bad Homburg) | Skin (ACA) | 4 | **+** | | **+** | | **-** | VF | |
|  | PKap | Germany (Tübingen) | Skin (ACA) | 2 | **+** | | **+** | | **+** | VF | |
|  | PKo^b^ | Germany | Skin, EM | 7 | **-** | | **-** | | **-** | SC from RM | |
|  | PKo^b^ | Germany (Munich) | Skin (EM) | 10 | **+** | | **+** | | **+** | VF | |
|  | PKr | Germany (Munich) | CSF (NB) | 2 | **+** | | **+** | | **+** | VF | |
|  | PObf | Germany (Bad Mertengheim) | Skin | 1 | **-** | | **-** | | **+** | VF | |
|  | PWe | Germany (Munich) | Skin (ACA) | 2 | **-** | | **-** | | **-** | VF | |
|  | R-IP3 | Russia | *I. persulcatus* | <10 | **+** | | **+** | | **+** | SC from PR | |
|  | R-IP21 ("IP21") | Russia | *I. persulcatus* | <10 | **-** | | **+** | | **+** | SC from RM | |
|  | TDe | Germany (unknown) | *I. ricinus* | 7 | **+** | | **+** | | **-** | VF | |
|  | T10 | Germany (Munich) | *I. ricinus* | <10 | **+** | | **+** | | **-** | VF | |
|  | UM01 | Sweden | Skin (EM) | <10 | **-** | | **+** | | **+** | SC from RM | |
|  | UO1 | Sweden | Skin (EM) | <10 | **-** | | **-** | | **-** | SC from RM | |
|  | VS461 | Switzerland | *I. ricinus* | <10 | **-** | | **-** | | **-** | SC from RM | |
|  | 61BV3 | Germany (Berlin) | *I. ricinus* | <10 | **+** | | **+** | | **-** | AS | |
|  | 97B3 | Germany (Berlin) | *I. ricinus* | <10 | **-** | | **-** | | **+** | AS | |
| ***B. spielmanii***  **(rare pathogen, Eurasia)** | Anzic | Slovenia | Skin (EM) | Unknown | **-** | | **-** | | **-** | FS | |
|  | A14S | The Netherlands | Skin | Low | **+** | | **+** | | **+** | AvD | |
|  | Isau2 | Germany (Munich) | *I. ricinus* | 2 | **-** | | **-** | | **-** | VF | |
|  | PHap | Germany (Munich) | Skin (EM) | 2 | **+** | | **+** | | **+** | VF | |
|  | PMai | Germany (Munich) | Skin (EM) | 7 | **+** | | **+** | | **+** | VF | |
|  | PMEW | Germany (Munich) | Skin (EM) | 5 | **+** | | **+** | | **+** | VF | |
|  | PSigII | Germany (Munich) | Skin (ACA) | 7 | **+** | | **+** | | **+** | VF | |
| ***B. bavariensis***  **(rare pathogen, Eurasia)** | PBaeI | Germany (Munich) | Skin (EM) | Low | **-** | | **-** | | **-** | GM from VF^1^ | |
|  | PBi | Germany (Ingolstadt) | CSF (NB) | 5 | **-** | | **-** | | **-** | VF | |
|  | PBN | Germany (Munich) | CSF | Low | **-** | | **-** | | **-** | GM from VF^1^ | |
|  | PFin | Germany (Munich) | CSF (NB) | 8 | **-** | | **-** | | **-** | VF | |
|  | PFlk | Germany (Munich) | CSF (EM & NB) | Low | **-** | | **-** | | **-** | GM from VF^1^ | |
|  | PHoe | Germany (Munich) | CSF (NB) | Low | **-** | | **-** | | **-** | GM from VF^1^ | |
|  | PNi | Germany (Halle) | Skin (EM) | 6 | **-** | | **-** | | **-** | VF | |
|  | POb | Germany (Munich) | Skin (EM) | Low | **-** | | **-** | | **-** | GM from VF^1^ | |
|  | PRab | Austria (Villach) | Hand joint synovia | Low | **-** | | **-** | | **-** | GM from VF^1^ | |
|  | PRof | Germany (Munich) | Skin (ACA) | 3 | **-** | | **-** | | **-** | VF | |
|  | PScf | Germany (Munich) | CSF (NB) | Low | **-** | | **-** | | **-** | GM from VF^1^ | |
|  | PTrob | Slovenia | Skin | Low | **-** | | **-** | | **-** | FS | |
| ***B. valaisiana***  **(probable pathogen, Eurasia)** | IPT29 | France (Meuse) | *I. ricinus* (N) | Unknown | **-** | | **+** | | **-** | IP | |
|  | IPT31 | France (Meuse) | *I. ricinus* (F) | Unknown | **-** | | **+** | | **-** | IP | |
|  | IPT33 | France (Meuse) | *I. ricinus* (N) | Unknown | **-** | | **+** | | **-** | IP | |
|  | IPT47 | France (Alsace) | *I. ricinus* (F) | Unknown | **-** | | **+** | | **-** | IP | |
|  | IPT85 | France (Alsace) | *I. ricinus* (M) | Unknown | **-** | | **+** | | **-** | IP | |
|  | IPT102 | France (Auvergne) | *I. ricinus* (F) | Unknown | **-** | | **+** | | **-** | IP | |
|  | IPT111 | France (Alsace) | *I. ricinus* (F) | Unknown | **-** | | **+** | | **-** | IP | |
|  | IPT121 | France (Alsace) | *I. ricinus* (F) | Unknown | **-** | | **+** | | **-** | IP | |
|  | IPT144 | France (Limousin) | *I. ricinus* (F) | Unknown | **+** | | **+** | | **+** | IP | |
|  | IPT163 | France (Auvergne) | *I. ricinus* (F) | Unknown | **-** | | **+** | | **-** | IP | |
|  | IPT174 | France (Auvergne) | *I. ricinus* (F) | Unknown | **-** | | **+** | | **-** | IP | |
|  | IPT177 | France (Limousin) | *I. ricinus* (F) | Unknown | **+** | | **+** | | **+** | IP | |
|  | IPT184 | France (Limousin) | *I. ricinus* (M) | Unknown | **-** | | **-** | | **-** | IP | |
|  | IPT186 | France (Limousin) | *I. ricinus* (F) | Unknown | **-** | | **+** | | **-** | IP | |
|  | IPT187 | France (Limousin) | *I. ricinus* (F) | Unknown | **-** | | **-** | | **-** | IP | |
|  | IPT188 | France (Normandie) | *I. ricinus* (M) | Unknown | **-** | | **+** | | **-** | IP | |
|  | VS116 | Switzerland | *I. ricinus* | 11 | **+** | | **+** | | **+** | SC from RM | |
|  | 10MT | Korea | *I*. *nipponensis* | <10 | **-** | | **-** | | **-** | SC from RM | |
|  | 61104BT | UK (Bath) | *I. ricinus* | 0 (not cultured) | **-** | | **-** | | **-** | GM | |
|  | 61214BT | UK (Bath) | *I. ricinus* | 0 (not cultured) | **-** | | **-** | | **-** | GM | |
|  | 61306L | UK (Bath) | *I. ricinus* | 0 (not cultured) | **-** | | **-** | | **-** | GM | |
| ***B. lusitaniae***  **(probable pathogen, Eurasia)** | Poti B2 | Portugal | *I. ricinus* | 7 | **-** | | **+** | | **+** | IP | |
|  | Poti B3 | Portugal | *I. ricinus* | 5 | **-** | | **+** | | **+** | IP | |
