# Supplementary material for: Identification of the minimal cytolytic unit for streptolysin S and an expansion of the toxin family
Source: BMC Microbiol. 2015 Jul 24;15:141. doi: 10.1186/s12866-015-0464-y (PMC4513790; doi:10.1186/s12866-015-0464-y)
Supplement: Additional file 7: Figure S4. — Nucleotide alignments of Bor TOMM biosynthetic genes. BorB (A), borC (B) and borD (C) from Borrelia afzelii PKo (Bafz), B. spielmanii A14S (Bspi), and B. valaisiana VS116 (Bval) were aligned using ClustalW. This phylogenetic data is deposited in the Dryad Digital Repository, doi:10.5061/dryad.d4863. Sites where PCR screening primers annealed are underlined. The primers used to detect borB/C/D (Table 1 and Additional file 6: Table S3) were based on bvalB/C/D from B. valaisiana VS116 (Additional file 8: Table S4). The number of mismatches per primer when compared to the two other sequenced examples of the gene is indicated in parentheses for each. [file 12866_2015_464_MOESM7_ESM.docx]

**Supplementary Figure 4**

A

***borB* (5’ mismatches = 3; 3’ mismatches = 3)**

BafzB ATGGTAAAAAATGACAATGAAAGTTCTGAAGTTTCTAATATGAAGCTATCAGGGATGTAT 60

BspiB ATGATAAAAAATGATAATGAAAGTTTTGAAGTTTCTAATATGAAGCTATCAGGGATGTAT 60

BvalB **ATGGTAAAAAATGATAATGAAAGTTCTGAAGT**TTTTAATATGAAGCTATCAGGGATGTAT 60

*** ********** ********** ******** *************************

BafzB GAAATAAATAATGTTTCAAGAAGTGTTTTTAATAGCAATATCATTGCTAATCCTTCTAAT 120

BspiB GAAATAAATAATATTTCAAGAAGTGTGTTTAATAGCAATATTATTGCTAATCCTTCTAAT 120

BvalB GAAATAAATAATGTTTCAAGAAGTGTGTTTAATAGCAATATTATTGCTATTCCTTCTAAT 120

************ ************* ************** ******* **********

BafzB ATTAGAATTCAAGCATCTTTATTTGATAAAGATATAGAAAGCTTGACTCTTAATTATTTA 180

BspiB ATGAGAATTCAAGCATCTTTGTTTGATAAAGATACAGAAAGTTTGACTCTTAATTATTTA 180

BvalB ATTAGAATTAAAGCATCTTTATTTGACAAAGATGCAGAAAGTTTGACTCTTAATTATTTA 180

** ****** ********** ***** ****** ****** ******************

BafzB TTAAATTTTTCTGAAATTAAAAAATTTTATTTTAATGAAAATATTTCTAAATTTTTGCAA 240

BspiB TTAAATTTTTCTGAAATTAAAAAATTTTATTTTAATGAAAATATTTCTCAATTTTTGCAA 240

BvalB TTAAATTTTTCTGAAATTAAAAAATTTTATTTTAATGAAAATATTTCTAAATTTTTGCAA 240

************************************************ ***********

BafzB AACCCCGCTGCTATTGCAAATATATCTAATAGAGAATATCGCGAATTTGACGA-GA---- 295

BspiB AATTCCGCTGCTATTGCAAATATATCTAACAGAGAATATCGTGAATTTAATGA-GA---- 295

BvalB GATCCCGCTGCTATTGCAAATATATCTAACAGAGAATATCGTGAATTTGATGATGATGAT 300

* ************************* *********** ****** * ** **

BafzB -ATGTAATTTATTTGCCTAAAGTTAAAAGACTTAAAATTAAACTTGAAGATGCTTTACTT 354

BspiB -ATGTAATTTATTTGCCTAAAGTTAAAAGACTCAAAATTAAACTTGAAGATGCTTTGCTT 354

BvalB GATGTAGTTTTTTTACCTAAAGTTAAAAGACTTAAAATTAAACTTGAAGATGCGTTGCTT 360

***** *** *** ***************** ******************** ** ***

BafzB AAAAGGAAATCTTCTCGAAAGTTTAGAAATCAATATTTAAATTTGGTGGATTTATCGACG 414

BspiB AAAAGAAAATCTTCTAGAAAGTTTAAAAATCAATATTTAAATTTGGTAGATTTGTCTACG 414

BvalB AAAAGAAAGTCTTATAGAAAGTTTAAAAATCAATATTTAAATTTGGTGGATTTATCGACG 420

***** ** **** * ********* ********************* ***** ** ***

BafzB CTTCTTTATTATGCTGCTGGTGATGTTCGATATGATTATGTAGAATTTGGAAATTCAAAA 474

BspiB CTTCTTTATTATTCTGCTGGTGATGTTAGATATGATTATGTAGAATTTGGAAATTCAAAA 474

BvalB CTTCTTTATTATGCTGCTGGCGATGTTCGATATGATTATGTAGAATTTGGAAATTCAAAA 480

************ ******* ****** ********************************

BafzB TTTAGGCAATCTAGAAAATCTTATCCATCAGGAGGCGGAATGTATCCGATAGATATTTAT 534

BspiB TTTAGGCAATCTAGAAAGACTTATCCATCAGGAGGAGGAATGTATCCTATAGATATTTAT 534

BvalB CTTAGGCAATCTAGGAAGCCTTATCCATCAGGGGGAGGAATGTATCCTATAGATATTTAT 540

************* ** ************* ** *********** ************

BafzB TTTTACGCAAATAATGTTGAAAAGCTTGATAAAGGTTTTTATTTATATCAATCTTCAAAT 594

BspiB TTTTACGCAAATAATGTTGAAAAGCTAGATAAAGGTTTTTATTTATATCAATCTTCAAAT 594

BvalB TTTTATGTAAATAATGTTGAAAAGCTTGATAAAGGTTTTTATTTATATCAATCTTCAAAT 600

***** * ****************** *********************************

BafzB AATTCAATTGTTAAGATCAATATTAGTTCGATTAAGATTGAAAATTTACTTACTATTTCG 654

BspiB AATTCAATTGTTAAGATTAATATAAGTTCGATTAAGATTGAAAATTTACTTAATATTTCG 654

BvalB AATTCAATTGTTAAGATCAATATTGGTTCGATTAAGATTGAAAATTTACTTACTATTTCG 660

***************** ***** *************************** *******

BafzB TTTTTTGAAGGTTCTTCTAATTTTAATATTGCTTTCTTTTTTGTTTATAGATATGAGGTT 714

BspiB TTTTTTGAGGGTTCTTCTAATTTTAATGTTGCTATCTTTTTTGTTTATAGATATGAGGTT 714

BvalB TTTTTTGAAGGTTCTTCTAATTTTAATATTGCTTTCTTTTTTGTTTATAGATATGAGGTT 720

******** ****************** ***** **************************

BafzB AATTATTTAAAGTATTCAGAAATGAGCTTGTCTTTTGCTTTTATAGAGCTAGGTGCTATT 774

BspiB AATTATTTGAAGTATTCAGAAATGAGTTTGTCTTTTACTTTTATAGAGTTAGGTGCTATT 774

BvalB AATTATTTAAAGTATTCAGAGATGAGTTTGTCTTTTGCTTTTATAGAGCTAGGCGCTATT 780

******** *********** ***** ********* *********** **** ******

BafzB CATCAGAATTTTTCTTTGGTTTCTGCTGCTTTGAATTTGGGGTATTGCAGTTGGGGGGGA 834

BspiB CATCAAAATTTTTCTTTGGTTTCTACTGATTTAAATCTGGGATATTGTGTCTGGGGAGGA 834

BvalB CATCAAAATTTTTCTTTAGTTTCTACTGCTTTGAATTTGGGATATTGTAGTTGGGGAGGG 840

***** *********** ****** *** *** *** **** ***** ***** **

BafzB TTTGATAAATCAGAGTCAGAAAAGTATTTAAAGCTAGATGGAATTAGTGAGCATGTAGTT 894

BspiB TTTGATAAGCCAGAGCTGGAAAAGTGTTTAAAGCTAGATGGAATTAGTGAACATGTAGTT 894

BvalB TTTGATAAACCAGAGCTAGAAAAGTGCTTAAAGTTAGATGGAATTAGTGAACATGTAGTT 900

******** ***** ******* ****** **************** *********

BafzB TCAGCTGCTATTTTAGGGAATGTTTAA 921

BspiB TCAGCTTCTATTTTAGGAAATGTTTAA 921

BvalB T**CAGCTGCTATTTTAGGGAATGTCTAA** 927

****** ********** ***** ***

B

***borC* (5’ mismatches = 2; 3’ mismatches = 1)**

BafzC ATGAATAATGGTTTATATTATTTTTCAGATAATGTGAGAGTTTTAAAAGAATCCAAAGGC 60

BspiC ATGAATAATGGTTTATATTATTTTTCAGATAATGTGAGAGTTTTAAAAGAATCCAAAGGC 60

BvalC **ATGAATAATGGTTTGTATTATTTTTCAGATAATGTAAG**AGTTTTAAAAGAATCCAAAGGG 60

************** ******************** ***********************

BafzC TTTTTGATTATTAGGAAGGGAGTTTGGAATTATGAGGATTTGATAATTTCTATTGAAGAT 120

BspiC TTTTTGATTGTTAGGAAGGGAGTTTGGAATTATGAGGATCTAACAATTTCTATTGAAGAT 120

BvalC TTTTTGATTGTTAGGAAGGGGGTTTGGAATTATGAGGACCTGATAATTTCTATTGAAGAT 120

********* ********** ***************** * * ****************

BafzC TCTTATATTAATGAATTGAGCAAAGTTTTTAATTCTTTATCTTCTAATGAGGGAATTGAT 180

BspiC TCTTATATTAATGAATTGAGCAAAGTTTTTAATTCTTTGTCTTCTGATGAAGGAATTGAT 180

BvalC TCTTATATTAATGAATTGAGCAAAGTTTTTAATTCTTTGTCTTCTGATGAGGGAATTGAT 180

************************************** ****** **** *********

BafzC TTGATTCAAATAAAAAATGATAGGGTTAAAAATATTGTTAAAGAATTAATTAATTCTGGA 240

BspiC TTGATTCAAATAAAAAATGATAAGGTCAAAAATATTGTTAAAGAATTAATTAATTCTGGT 240

BvalC TTAATTCAAATAAAAAACAATAAGGTTAAAAATATTGTTAAAGAATTAATTAATTCTGGG 240

** ************** *** *** ********************************

BafzC TTTGTTCATTTGAATGAGTTTAGGAAAAATAATAGATTTTTAAATTATTTATATCTTGGT 300

BspiC TTTGTTCATTTGAATGAGTTTGGAGAAAATAATAGATTTTTAAATTATTTATATCTTGGT 300

BvalC TTTGTTCATTTGAATGAGTTCAGAATTAATAATAGATTTTTAAATTATTTATATTTTGGG 300

******************** * *************************** ****

BafzC CAATATTTAGATAGTGTTCCCCAAGAGAATCCTTATTTGCTGATTTCAGATGATAGCAGC 360

BspiC CAATATTTAGAAGTTGCTCCCCAAGAGAATCCTTATTTATTGATTTCAGATGATAATAGT 360

BvalC CAATATTTGGAAGTTGTTCCCCAAGAGAATCCTTATTTGCTGATTTCAGATGATAGTAGT 360

******** ** ** ********************* *************** **

BafzC TTGATTAATGTTTTAGAAACCACTGCTAAATCGTTTGATTTAAAGTATGAGTTTTTGAGT 420

BspiC TTGATTAATATTTTAGAAACAACCGCTAAATCGTTTAATTTAAAGTATGAGCTTTTGAGT 420

BvalC TTGATTAATATTCTAGAAACTACTGCTAAATCGTTTGATTTAAAGTATGAGCTTTTGAGT 420

********* ** ******* ** ************ ************** ********

BafzC CAGGAAAACTTGGAATTTTTAAAAACACCTGTTTTTTTAAATAAATTGGATTTAATAAAT 480

BspiC CGGGAAAACTTGGAATTTTTAGAAACACCTATTTTTTTAAATAAATTAGATTTAATAAAT 480

BvalC CATGAAAACTTGGAATTTTTAAAAACACCTGTTTTTTTAAATAAATTGGATTTAATGCGG 480

* ****************** ******** **************** ********

BafzC TATTCAAAAAGTTTAGACCATTTTAGATCCAAATTTATGCAATTTGAAGGGGTTATTCTG 540

BspiC TATTCAAAAAGTTTAGACCATTTTAGATCGAAATTTATGCCATTTGAAGGGGTTATTCTA 540

BvalC TATTCAAAAAGTTTAGATCATTTTAGATCCAAATTTATGCCATTTGAAGGGGTTATTATG 540

***************** *********** ********** **************** *

BafzC TTGTTGGGCAATTTGAATCCATTTTTACTTAGAAATATTAACAGAATTTTAATAGGACTT 600

BspiC TTATTGGGTAATTTGAATCCATTTTTACTTAGGAATATTAATAGAATTTTAATAGGACTT 600

BvalC TTGTTGGGTAATTTGAACCCATTTTTACTTAGAAATATTAATAGAATTTTAATAGGACTT 600

** ***** ******** ************** ******** ******************

BafzC AATAAACCAATTTTTTTTGGCACCATAGATGGTCCTTTCATGTTGATTACATGTTTAGAG 660

BspiC AATAAACCAATTTTTTTTGGTACCATAGATGGTCCTTTTATGTTGATTACATGTTTAGAG 660

BvalC AATAAACCAATTTTTTTTGGCACCATAGATGGGCCTTTTATGCTGATTACATGTTTAGAG 660

******************** *********** ***** *** *****************

BafzC CCCAAAAGAACAGCGTGTATTGAATGTTATGAGCAAAGGATAATTGCTAGGATGACGGAT 720

BspiC CCTAAAAGAACAGCATGTATTGAATGTTATGAGCAAAGGATAATTGCTAGGATGACAGAT 720

BvalC CCTAAAAGAACAGCATGTATTGAATGTTATGAACAAAGGATAATTGCTAGAATGACAGAT 720

** *********** ***************** ***************** ***** ***

BafzC CATGTACTTTATCATAGATTTAATAAGGAGTATGAAAAACACAAGAGGGATTCAAAGTTA 780

BspiC CATGTGCTTTATCATAGATTTAATAAGCAGTATGAAAAACATAAGAGGGATCCAAATTTA 780

BvalC CATGTACTTTATCATAGATTTAATAAGGAGTATGAAAAACATAAGAGGAACACAGAGTTA 780

***** ********************* ************* ****** * ** * ***

BafzC GTTTCTTTAGGGGATTTTTTAAGTCCAGCGTATTATCATATTGCTTACATTTTAGTTGGG 840

BspiC GTTTCTTCAGGGGATTTTTTAAGTCCTGCTTATTACCATATTGCTTACATTTTAGTTGGG 840

BvalC ATTTCTTCAGGAGATTTTTTAAGTCCAGCTTATTATCATATTGCTTACATTTTAGTTGGG 840

****** *** ************** ** ***** ************************

BafzC GATGCTTTTACTTTTTTTCAGGTTAAAACATCTAAACTTATGGGGAGAGCTTTGCATATT 900

BspiC GATGCTTTTACCTTTTTTCAGATTAAAACATCTAAGCTCATGGGAAGAGCTTTACATATT 900

BvalC GATGCTTTTACCTTTTTTCAGGTTAAAACATCTAAACTTATGGGGAGAGCTTTGCATATT 900

*********** ********* ************* ** ***** ******** ******

BafzC TATGTGCCTTTTTTTGAGTTCCAAAATCAAGATGTTTTGAGAATATCATCTTGTCCTGCT 960

BspiC TATGTACCTTTTTTTGAGTTTCAAAATCAAGATGTTTTGAGGATATCATCTTGTCCTGCT 960

BvalC TATGTGCCTTTTTTTGAATTCCAAAATCAAGATGTTTTGAGAATATCATCTTGTCCTGCT 960

***** *********** ** ******************** ******************

BafzC TGTGGATATTTGTCAACTTATAAATCAAGATGTTTGAATATTGAAACACAAAAGCTTGTT 1020

BspiC TGTGGATATTTGTCGACTTATAAATCAAGATGTTTGAATATTGAAACACAAAAACTTGTT 1020

BvalC TGTGGATATTTGTCGACTTATAAATCAAGATGTTTGAATATTGAAACTCA**AAAGCTTGTT** 1020

************** ******************************** ***** ******

BafzC TCAACATTAATTTCTAATTCTAATTAA 1047

BspiC TCAACATTAATTTCTAATTCTAATTAA 1047

BvalC **TCAACATTAATTTCTAATTCTAATTAA** 1047

***************************

C

***borD* (5’ mismatches = 7; 3’ mismatches = 3)**

BafzD ATGTTGAATTATTATCCTTATTCAAGTAAGCTCTATAGAGATCTTGTTTTTACTAATTCT 60

BspiD GTGTTTAATTATTATCCTTATTCAAGCAAGCTTTATAGAGATCTTGTTTTTACTAATTCT 60

BvalD **GTGATTAACTATTATCCTTATTCTAGTAAGCTTTATAGA**GATCTTGTTTTTACTAATTCT 60

** * ** ************** ** ***** ***************************

BafzD CCGGCTACAGGCATTGGTCCTTCGTTAGTTACATTGTTGCCTTTTCAAAAGGGATTGCCT 120

BspiD CCGGCAACAGGTATTGGTCCGTCTTTAGTTACATTATTGCCTTTTCAAAAGGGATTGCCT 120

BvalD CCGGCTACAGGTATTGGTCCAGCTTTAGTTACATTGTTACCCTTTCAAAAGGGATTGCCT 120

***** ***** ******** * *********** ** ** ******************

BafzD TTATTATATTCTTCTACATGTATTTTGCCTAATTATCACAAAATATTAATAGGAGAGACA 180

BspiD TTATTATATTCTTCTACATGTGTTTTACCTAATTACCATAAAATATTAATAGGGGAGACA 180

BvalD TTATTATATTCTTCTACATGTATTTTGCCCAATTATCATAAAATATTAATAGGAGAGGCA 180

********************* **** ** ***** ** ************** *** **

BafzD TGTAATATGGAATATCATATTTCTGGTTATGGGCGTTCTTATGAGGAGGCTATTACTAGA 240

BspiD TTTAATATGGAATATCATATTTCTGGTTATGGACGCTCTTATGAGGAGGCTATTACTAGA 240

BvalD TACAATATGGAATACCATATTTCTGGTTATGGGCGTTCTTATGAGGAGGCTATTACTAGA 240

* *********** ***************** ** ************************

BafzD TTACAAGGCGAAACTATTGAGAGATATTCTCTTTTGATGTCCAAAACTTTATTTCAAGAA 300

BspiD TTACAAGGCGAAACTATTGAGAGATATTCCCTTTTGATGTCCAAAACTTTATTTCAAGAA 300

BvalD TTGCAAGGTGAAACTATTGAGAGATATTCTCTTTTAATGTCTAAAACTTTATTTCAAGAA 300

** ***** ******************** ***** ***** ******************

BafzD GATTTTATTCTTTCTTCTAGAAAGGCCCTTTTAAATACCTCAAAGTATAAAGTTATGCCT 360

BspiD GATTTTATTCTTTCTTCTAGAAAGGAGCTTCTAAATACTTCAAAGTATAAGGTTATGCCT 360

BvalD GATTTTATTCTTTCTTCTAGAAAGTCTCTTCTAAATACTTCAAAGTATAAAGTTATGCCT 360

************************ *** ******* *********** *********

BafzD TTAGAATATAGAAATGTTTTTTCAGATTTAGATAGAAACTTTAATCTTCCTTATTCAAAA 420

BspiD TTGGAATATAGAAATGTTTTTTCAGATTTAGATAGAAATTGTAATCTTCCTTATTCAAAA 420

BvalD TTGGAGTATAGAAACGTTTTTTCAGATTTGGATAGAAATTTTAACCTTCCTTATACAAAA 420

** ** ******** ************** ******** * *** ********* *****

BafzD GTTAATGAGAGCGATGAAATTTATTGGATTTTATTGCCATCTTTAATTCATTCTAATGAA 480

BspiD GTTTATGAGAGCGATGAAATTTATTGGATTTTATTGCCATCCTTAATTCATTCCAATGAA 480

BvalD GTTTGTGAGAGTGATGAGATTTATTGGATTTTATTGCCATCTTTAATTCATTCTAATGAA 480

*** ****** ***** *********************** *********** ******

BafzD AAAACATGGGTTCCTTGTGATATGGTTTTTATGGGTATCCAGCAAGATTTTAAAATGCCA 540

BspiD AAAATATGGGTTCCTTGCGATATGATTTTTATGGGTATTCATCAGAATTTTAAAATGCCA 540

BvalD AAAATATGGGTCCCTTGCGATATGATTTTTATGGGTATCCATCAGAATTTTAAAATGCCA 540

**** ****** ***** ****** ************* ** ** **************

BafzD GCTTTTAGTACAGGAACAGCAGTTCATAGGACAGTGGAGATTGCACTTAGTAATGCAATA 600

BspiD ACTTTTAGTACAGGAACAGCAGTTCATAGGACAGTAGAGATTGCGCTTAGTAATGCAATA 600

BvalD ACTTTTAGTACAGGAACAGCAGTTCATAGGTCAGTAGAGATTGCACTTAGCAATGCAATA 600

***************************** **** ******** ***** *********

BafzD ATAGAGGTAATACAATTACATTGTTATATTTCTAATTGGTACGTAAAATCCAAAAGGCCT 660

BspiD ATAGAGGTAATACAATTACATTGTTATATTTCTAATTGGTACGTAAAATCCAAAAGGCCC 660

BvalD ATAGAGGTAATACAATTACATTGCTATATTTCTAATTGGTACATGAAATCTAAAAGGCCT 660

*********************** ****************** * ***** ********

BafzD GTGATTGACTGGAAAAGTAATAAATTTTTGAGAAAATCAATTAGTGATTTAGAACTTGAG 720

BspiD GTGATTGACTGGAAAAGTAATAAATTTTTGAGAAAATCAATTAGTGATTTAGAACTTGAA 720

BvalD GTGATTGACTGGAAAAGTAATAAATTTTTGAGAAAATCAATTGGTGATTTAGAACTTGAA 720

****************************************** ****************

BafzD TCCAATTTTGATTTAATAATTTTAGATTACTCTGATAGAGATTTGGGAATGCCAGTTTAT 780

BspiD TCCAATTTTGATTTAATAGTTTTAGATTATTCTGATAAAGATTTAGGAATGCCCGTTTAT 780

BvalD TCTTATTTTGATTTAGTTATTTTGGATTATTCTGATAGGGATTTGGGAATGCCAGTTTAT 780

** *********** * **** ***** ******* ***** ******** ******

BafzD GCAGCATTTTTAAGAAATAAGAAAAAATCGATTCCGTATTTAGTTTGTGGAATTCAAGGG 840

BspiD GCAGCATTCCTAAGAAATAAGAAAAAATCGATTCCATATTTAGTTTGTGGAATTCAAGGT 840

BvalD GCAGCATTCTTAAGAAATAAGAAAAAATCGATTCCATATTTAGTTTGTGGAATTCAAGGA 840

******** ************************* ***********************

BafzD GGCTTTAATAAAGAGTATGTTTTGTTAAGAGCCGTAGAAGAAGCCGCTGTGATTGCTCAA 900

BspiD GGCTTTAATAAAGAGTATGTTTTTTTAAGAGCTGTAGAAGAAGCTGCTGTAGTTGCTCAA 900

BvalD GGTTTTAATAAAGAGTATGTTTTGTTAAGAGCTGTAGAAGAAGCTGCTGTAGTTGCTCAG 900

** ******************** ******** *********** ***** *******

BafzD TCTTTACCTTTAATTTATTTTTTAAAGGCTAAAGAGATAAGCAAGTTAACATTAAATAGT 960

BspiD TCTTTGCCCCTAATTTATTTTTTTAAGGCTAAAGAAATAAGTAAGTTAACATTAAATAGT 960

BvalD TCTTTACCTTTAATTTATTTTTTTAAGGCTAGAGAGATAAGTAATTTAACATTAAATAGT 960

***** ** ************* ******* *** ***** ** ***************

BafzD CTAAGACATAGTTTCAATTTGGATGATAATTTTTTATATTACTCTAACTTAAATGAAATT 1020

BspiD CTAAGACACAGTTTTAATTTGGATGATAATTTTTTCTATTACTCCAATTTAAATGAAATT 1020

BvalD CTGAGGCACAGTTTTAATTTGGATGATAATTTTTTATATTATTCCAACCTGAATGAAATT 1020

** ** ** ***** ******************** ***** ** ** * *********

BafzD TCTTTAAAAGATTCTTTATTAAATTCTATTATAGATGATGAAACTAGATTAGAGATATTT 1080

BspiD TCTTTAAAAGATTCTATGTTAAATTCTATTATAGATAATGAAACTAGATTAGAGATATCT 1080

BvalD TCTTTAAAAGATTCTTTGTTGAATTCTATTATGGATAATGAAACTAGATTAGAGATATCT 1080

*************** * ** *********** *** ********************* *

BafzD TCTGAAGAGAACTTAGTTGAAAATGAATTGGATATGAGTTTAAATATTTTGAAGTCTGTA 1140

BspiD TCTGAAGAAAGCTTAATTAAAAATGAATTGGATATGAGCTTAAATATTTTGCGATCTGTA 1140

BvalD TCTGAAGAAAACTTAGTTGAAAATGAATTGAATATGAGTTTAAGTATTTTACGATCTGTA 1140

******** * **** ** *********** ******* **** ****** ******

BafzD AGTAAATATGCTGTTTACTGTGATATAACTCCCCCCGAATTAGAAAGTACATCTTTTAGG 1200

BspiD AGTAAATATGCTGTTTATTGCGATATAACTCCTCCCGAATTAGAAGGTAGATCTTTTAGA 1200

BvalD AGTAAATATGCTGTTTATTGTGATATAACTCCTCCTGAATTAGAAAGCACATCTTTTAAA 1200

***************** ** *********** ** ********* * * ********

BafzD GCTATAAGGATTCTTGTTCCTGAGTTGCTTAAAATGTGTTTTCCATTTCATCCCTTTGAT 1260

BspiD TCTATAAGGATTCTTGTTCCTGAATTGCTTAAGATGTGTTTTCCATTTCATCCCTTTGAT 1260

BvalD TCTATAAGGATTCTTGTTCCAGAGTTGCTTAAAATGTGTTTTCCATTTCACCCCTTTGAT 1260

******************* ** ******** ***************** *********

BafzD AAGCATCCATATTTTAAAAAGAAAGGAGAAATTTTAGATGGATATTTCCCACACCCAATA 1320

BspiD GAGCATCCATATTTTAAAAAGAAAGGAGAAATTTTAGATGAATATTTCCCACATCCAATA 1320

BvalD GAGCATCCATATTTTAAAAAGAAAGGAGAAATTTTAGAT**GAATATTACCCACACCCAATA** 1320

*************************************** ***** ****** ******

BafzD CCTTAG 1326

BspiD CCTTAG 1326

BvalD **CCTTAG** 1326

******
