# Supplementary material for: Identification of the minimal cytolytic unit for streptolysin S and an expansion of the toxin family
Source: BMC Microbiol. 2015 Jul 24;15:141. doi: 10.1186/s12866-015-0464-y (PMC4513790; doi:10.1186/s12866-015-0464-y)
Supplement: Additional file 8: Table S4. — Primers used in this study. In all cases, bvalA/B/C/D or BvalA/B/C/D refers to the relevant TOMM genes or proteins from B. valaisiana VS116. Restriction endonuclease recognition sites are underlined. Lower-case letters indicate bases targeted for mutagenesis. [file 12866_2015_464_MOESM8_ESM.docx]

**Supplementary Table 4**

| **Primer Name** | **Sequence (5’** → **3’)** | **Description** |
| --- | --- | --- |
| **PCR Screening** | | |
| BvalA-fwd | ATGTTTATTAGAAATAAACATTTTTTGACTAGC | Forward primer to amplify *borA* based on *bvalA* |
| BvalA-rev | TTATAGACTAAAGCTGCAACTACAAGTGC | Reverse primer to amplify *borA* based on *bvalA* |
| BvalB-fwd | ATGGTAAAAAATGATAATGAAAGTTCTGAAGT | Forward primer to amplify *borB* based on *bvalB* |
| BvalB-rev | TTAGACATTCCCTAAAATAGCAGCTG | Reverse primer to amplify *borB* based on *bvalB* |
| BvalC-fwd | ATGAATAATGGTTTGTATTATTTTTCAGATAATGTAAG | Forward primer to amplify *borC* based on *bvalC* |
| BvalC-rev | TTAATTAGAATTAGAAATTAATGTTGAAACAAGCTTT | Reverse primer to amplify *borC* based on *bvalC* |
| BvalD-fwd | GTGATTAACTATTATCCTTATTCTAGTAAGCTTTATAGA | Forward primer to amplify *borD* based on *bvalD* |
| BvalD-rev | CTAAGGTATTGGGTGTGGGTAATATTC | Reverse primer to amplify *borD* based on *bvalD* |
| **pDCerm Constructs** | | |
| BvalA-5'XbaI | AAATCTAGAATGTTTATTAGAAATAAACATTTTTTGAC | Forward primer to subclone *bvalA* from pIDTSMART construct |
| BvalA-3'BamHI | AAAGGATCCTTATAGACTAAAGCTGCAACTACAAGTG | Reverse primer to subclone *bvalA* from pIDTSMART construct |
| SagA-BvalA-5'XbaI | AAATCTAGAATGTTAAAATTTACTTCAAATATTTTAGCTAC | Forward primer to subclone *sagA-bvalA* from pIDTSMART construct |
| SagA-BvalA-3'BamHI | AAAGGATCCTTATAGACTAAAGCTGCAACTACAAGTGCAAC | Reverse primer to subclone *sagA-bvalA* from pIDTSMART construct |
| sagA^1-50^For | GAAGTTATACGtaAGGTAAATAAGGATCCTGATCTTCAGAA | Forward primer for incorporation of stop codon at position corresponding to P51 in *sagA* |
| sagA^1-50^Rev | CCTTATTTACCTtaCGTATAACTTCCGCTACCACCTTGAGA | Reverse primer for incorporation of stop codon at position corresponding to P51 in *sagA* |
| sagA^1-44^For | ATTCTCAAGGTtaaAGCGGAAGTTATACGCCAGGTAAATAA | Forward primer for incorporation of stop codon at position corresponding to G45 in *sagA* |
| sagA^1-44^Rev | TAACTTCCGCTttaACCTTGAGAATTACCACTTCCAGTAGC | Reverse primer for incorporation of stop codon at position corresponding to G45 in *sagA* |
| sagA^1-42^For | GTGGTAATTCTtAAGGTGGTAGCGGAAGTTATACGCCAGGT | Forward primer for incorporation of stop codon at position corresponding to Q43 in *sagA* |
| sagA^1-42^Rev | CCGCTACCACCTTaAGAATTACCACTTCCAGTAGCAATTGA | Reverse primer for incorporation of stop codon at position corresponding to Q43 in *sagA* |
| sagA^1-40^For | CTGGAAGTGGTtAaTCTCAAGGTGGTAGCGGAAGTTATACG | Forward primer for incorporation of stop codon at position corresponding to N41 in *sagA* |
| sagA^1-40^Rev | CCACCTTGAGAtTaACCACTTCCAGTAGCAATTGAGAAGCA | Reverse primer for incorporation of stop codon at position corresponding to N41 in *sagA* |
| sagA^1-38^For | TTGCTACTGGAtaaGGTAATTCTCAAGGTGGTAGCGGAAGT | Forward primer for incorporation of stop codon at position corresponding to S39 in *sagA* |
| sagA^1-38^Rev | TGAGAATTACCttaTCCAGTAGCAATTGAGAAGCAACAAGT | Reverse primer for incorporation of stop codon at position corresponding to S39 in *sagA* |
| sagA^1-37^For | CAATTGCTACTtaAAGTGGTAATTCTCAAGGTGGTAGCGGA | Forward primer for incorporation of stop codon at position corresponding to G38 in *sagA* |
| sagA^1-37^Rev | GAATTACCACTTtaAGTAGCAATTGAGAAGCAACAAGTAGT | Reverse primer for incorporation of stop codon at position corresponding to G38 in *sagA* |
| sagA^1-36^For | TCTCAATTGCTtaaGGAAGTGGTAATTCTCAAGGTGGTAGC | Forward primer for incorporation of stop codon at position corresponding to T37 in *sagA* |
| sagA^1-36^Rev | TTACCACTTCCttaAGCAATTGAGAAGCAACAAGTAGTACA | Reverse primer for incorporation of stop codon at position corresponding to T37 in *sagA* |
| sagA^1-35^For | GCTTCTCAATTtaaACTGGAAGTGGTAATTCTCAAGGTGGT | Forward primer for incorporation of stop codon at position corresponding to A36 in *sagA* |
| sagA^1-35^Rev | CCACTTCCAGTttaAATTGAGAAGCAACAAGTAGTACAGCA | Reverse primer for incorporation of stop codon at position corresponding to A36 in *sagA* |
| sagA^1-34^For | GTTGCTTCTCAtaaGCTACTGGAAGTGGTAATTCTCAAGGT | Forward primer for incorporation of stop codon at position corresponding to I35 in *sagA* |
| sagA^1-34^Rev | CTTCCAGTAGCttaTGAGAAGCAACAAGTAGTACAGCAGCA | Reverse primer for incorporation of stop codon at position corresponding to I35 in *sagA* |
| sagA^1-33^For | CTTGTTGCTTCtaaATTGCTACTGGAAGTGGTAATTCTCAA | Forward primer for incorporation of stop codon at position corresponding to S34 in *sagA* |
| sagA^1-33^Rev | CCAGTAGCAATttaGAAGCAACAAGTAGTACAGCAGCAACA | Reverse primer for incorporation of stop codon at position corresponding to S34 in *sagA* |
| sagA-bvalA+A_For | GCTTTAGTCTAgctTAAGGATCCTGATCTTCAGAACTTAAG | Forward primer for appending alanine codon after position corresponding to L36 in *sagA*-*bvalA* |
| sagA-bvalA+A_Rev | CAGGATCCTTAagcTAGACTAAAGCTGCAACTACAAGTGCA | Reverse primer for appending alanine codon after position corresponding to L36 in *sagA*-*bvalA* |
| sagA-bvalA-S27CFor | GCTGCTGTTGTtgcTGCACTTGTAGTTGCAGCTTTAGTCTA | Forward primer for substitution with cysteine codon at position corresponding to S27 in *sagA*-*bvalA* |
| sagA-bvalA-S27CRev | CTACAAGTGCAgcaACAACAGCAGCCTCCAGGAGCAACTTG | Reverse primer for substitution with cysteine codon at position corresponding to S27 in *sagA*-*bvalA* |
| pDC_SeqMCS_F | GGGAGGAAATAAAGCGCTAGG | Forward primer for verification of site-directed mutants of *sagA* by DNA sequencing |
| ***S. pyogenes* M1 *ΔsagA* Transformant Confirmation** | | |
| pDC_SeqMCS_F | GGGAGGAAATAAAGCGCTAGG | Forward primer to detect pDCerm containing gene of interest |
| pDCerm_PCR_R | ACAAGTAACCAGTGACTGCCG | Reverse primer to detect pDCerm containing gene of interest |
| SagB_F | ATGTCATTTTTTACAAAGGAACAA | Forward primer to detect *sagB* gene |
| SagB_R | ATTGACGATGACTTCTTCG | Reverse primer to detect *sagB* gene |
| spy1258F | AAAGACCGCCTTAACCACCT | Forward primer to detect gene unique to *S. pyogenes* ([Liu et al., 2005](#_ENREF_1)) |
| spy1258R | TGGCAAGGTAAACTTCTAAAGCA | Reverse primer to detect gene unique to *S. pyogenes* ([Liu et al., 2005](#_ENREF_1)) |
| **pET28b-MBP Constructs** | | |
| BvalA-5'BamHI | AAAAGGATCCATGTTTATTAGAAATAAAC | Forward primer to subclone *bvalA* from pIDTSMART construct |
| BvalA-3'NotI | AAAAGCGGCCGCTTATAGACTAAAGCTGC | Reverse primer to subclone *bvalA* from pIDTSMART construct |
| SagA-BvalA-5'BamHI | AAAAGGATCCATGTTAAAATTTACTTCAAA | Forward primer to subclone *sagA-bvalA* from pIDTSMART construct |
| SagA-BvalA-3'NotI | AAAAGCGGCCGCTTATAGACTAAAGCTGCAAC | Reverse primer to subclone *sagA-bvalA* from pIDTSMART construct |
| sagA^1-35^For | GCTTCTCAATTtaaACTGGAAGTGGTAATTCTCAAGGTGGT | Forward primer for incorporation of stop codon at position corresponding to A36 in *sagA* |
| sagA^1-35^Rev | CCACTTCCAGTttaAATTGAGAAGCAACAAGTAGTACAGCA | Reverse primer for incorporation of stop codon at position corresponding to A36 in *sagA* |
