# Supplementary material for: Identification of the minimal cytolytic unit for streptolysin S and an expansion of the toxin family
Source: BMC Microbiol. 2015 Jul 24;15:141. doi: 10.1186/s12866-015-0464-y (PMC4513790; doi:10.1186/s12866-015-0464-y)
Supplement: Additional file 10: Table S5. — Strains and plasmids used in this study. In all cases, bvalA/BvalA refers to the TOMM gene/peptide from B. valaisiana VS116. The sequences of the relevant precursor peptides are shown in Figs. 2A and 5A. [file 12866_2015_464_MOESM10_ESM.docx]

**Supplementary Table 5**

| **Strain/Plasmid** | **Relevant characteristics/Uses** | **References** |
| --- | --- | --- |
| **Strains** | | |
| *Escherichia coli* DH5α | Plasmid construction/maintenance | Lab stock |
| *Escherichia coli* BL21(DE3)-RIPL | Protein overexpression for purification | Lab stock |
| *Streptococcus pyogenes* (GAS) M1 | Group A Streptococcus | Lab stock |
| *Streptococcus pyogenes* (GAS) M1 Δ*sagA* | Group A Streptococcus; *sagA* allelic exchange mutant of *S. pyogenes* M1 | [21] |
| **Plasmids** | | |
| pDCerm-empty | Empty GAS expression vector; Erm^R^ | [21] |
| pDCerm-*sagA* | Constitutive expression of SagA wild-type precursor peptide; compliments GAS M1 *ΔsagA* | [21] |
| pDCerm-*sagA*^1-50^ | Constitutive expression of SagA^1-50^ truncation | This study |
| pDCerm-*sagA*^1-44^ | Constitutive expression of SagA^1-44^ truncation | This study |
| pDCerm-*sagA*^1-42^ | Constitutive expression of SagA^1-42^ truncation | This study |
| pDCerm-*sagA*^1-40^ | Constitutive expression of SagA^1-40^ truncation | This study |
| pDCerm-*sagA*^1-38^ | Constitutive expression of SagA^1-38^ truncation | This study |
| pDCerm-*sagA*^1-37^ | Constitutive expression of SagA^1-37^ truncation | This study |
| pDCerm-*sagA*^1-36^ | Constitutive expression of SagA^1-36^ truncation | This study |
| pDCerm-*sagA*^1-35^ | Constitutive expression of SagA^1-35^ truncation as BvalA mimic | This study |
| pDCerm-*sagA*^1-34^ | Constitutive expression of SagA^1-34^ truncation | This study |
| pDCerm-*sagA*^1-33^ | Constitutive expression of SagA^1-33^ truncation | This study |
| pDCerm-*bvalA* | Constitutive expression of BvalA wild-type precursor peptide | This study |
| pDCerm-*sagA-bvalA* | Constitutive expression of SagA^1-23^ fused to BvalA^25-37^ to yield chimeric precursor peptide consisting of SagA leader with BvalA core | This study |
| pDCerm-*sagA*-*bvalA*+A | Constitutive expression of SagA-BvalA chimera with alanine residue appended after terminal leucine residue | This study |
| pDCerm-*sagA*-*bvalA*-S27C | Constitutive expression of SagA-BvalA chimera with S27C substitution | This study |
| pET28b-MBP | Empty *E. coli* vector for high heterologous expression of proteins; Kan^R^ | [6] |
| pET28b-MBP-*sagA* | Overexpression of SagA plus N-terminal MBP tag | [6] |
| pET28b-MBP-*sagA*^1-35^ | Overexpression of SagA^1-35^ truncation as BvalA mimic plus N-terminal MBP tag | This study |
| pET28b-MBP-*bvalA* | Overexpression of BvalA plus N-terminal MBP tag | This study |
| pET28b-MBP-*sagA-bvalA* | Overexpression of SagA^1-23^ fused to BvalA^25-37^ to yield chimeric precursor peptide consisting of SagA leader with BvalA core plus N-terminal MBP tag | This study |
| pET28b-MBP-*sagA*-C32A | Overexpression of SagA precursor peptide with C32A substitution plus N-terminal MBP tag | [7] |
| pET28b-MBP-*sagB* | Overexpression of SagB dehydrogenase plus N-terminal MBP tag | [6] |
| pET28b-MBP-*sagC* | Overexpression of SagC component of cyclodehydratase plus N-terminal MBP tag | [6] |
| pET28b-MBP-*sagD* | Overexpression of SagD component of cyclodehydratase plus N-terminal MBP tag | [6] |
