# Supplementary material for: Identification of the minimal cytolytic unit for streptolysin S and an expansion of the toxin family
Source: BMC Microbiol. 2015 Jul 24;15:141. doi: 10.1186/s12866-015-0464-y (PMC4513790; doi:10.1186/s12866-015-0464-y)
Supplement: Additional file 4: Table S2. — List of accession identifiers for D proteins of putative TOMM cytolysin clusters. The genus, species, strain, and GenBank accession identifiers are given for all D proteins used in the phylogenetic analysis (Fig. 3), with inherent duplications from GenBank RefSeq annotations deleted. [file 12866_2015_464_MOESM4_ESM.pdf]

Organism	Accession
Bacillus coagulans	WP_017550911.1
Borrelia afzelii	WP_014486303.1
Borrelia afzelii PKo	YP_005592169.1
Borrelia garinii	WP_029362437.1
Borrelia spielmanii	WP_012665778.1
Borrelia spielmanii A14S	ACN53165.1
Borrelia valaisiana	WP_012665421.1
Borrelia valaisiana VS116	YP_002642260.1
Brachyspira alvinipulli	WP_028330607.1
Brachyspira hampsonii	WP_008723840.1
Brachyspira hampsonii 30446	EKV56787.1
Brachyspira hampsonii 30599	ELV05843.1
Brachyspira hyodysenteriae	WP_012671839.1
Brachyspira hyodysenteriae WA1	YP_002722513.1
Brachyspira innocens	WP_020004891.1
Brachyspira intermedia	WP_014488740.1
Brachyspira intermedia PWS/A	AEM22928.1
Brachyspira murdochii	WP_013113517.1
Brachyspira murdochii DSM 12563	YP_003633290.1
Brachyspira pilosicoli	WP_014933816.1
Brachyspira pilosicoli 95/1000	YP_003785416.1
Brachyspira pilosicoli B2904	AFR69796.1
Brachyspira pilosicoli P43/6/78	AGA67271.1
Brachyspira pilosicoli WesB	CCG57701.1
Catelliglobosispora koreensis	WP_026208092.1
Clostridium algidicarnis	WP_029453539.1
Clostridium botulinum	KGO12978.1
Clostridium botulinum A str. ATCC 19397	YP_001382880.1
Clostridium botulinum A str. ATCC 3502	YP_001253033.1
Clostridium botulinum A str. Hall	ABS36674.1
Clostridium botulinum A2 str. Kyoto	YP_002802818.1
Clostridium botulinum A2B3 87	KEI98503.1
Clostridium botulinum A3 str. Loch Maree	YP_001785837.1
Clostridium botulinum Af84	EPS53302.1
Clostridium botulinum B str. Osaka05	GAE00980.1
Clostridium botulinum B1 str. Okra	YP_001780137.1
Clostridium botulinum Ba4 str. 657	ACQ55104.1
Clostridium botulinum Bf	EDT87439.1
Clostridium botulinum CFSAN001627	EKN42007.1
Clostridium botulinum CFSAN001628	EKX80885.1
Clostridium botulinum F str. Langeland	ABS40086.1
Clostridium botulinum H04402 065	YP_005677074.1
Clostridium botulinum NCTC 2916	EDT83567.1
Clostridium papyrosolvens DSM 2782	WP_004620508.1
Clostridium sporogenes	KCZ68546.1
Clostridium sporogenes ATCC 15579	EDU38309.1
Clostridium sporogenes PA 3679	EHN15787.1
Enterococcus caccae	WP_010772870.1
Enterococcus caccae ATCC BAA-1240	EOL43456.1
Enterococcus casseliflavus	WP_010748138.1
Enterococcus durans	WP_016176860.1
Enterococcus durans ATCC 6056	EOT25568.1
Enterococcus faecalis	WP_010829081.1
Enterococcus faecalis 02-MB-BW-10	EPH79378.1
Enterococcus faecalis 20.SD.W.06	EPI05532.1
Enterococcus faecalis ATCC 10100	EOK06979.1
Enterococcus faecalis ATCC 27275	EOJ36344.1
Enterococcus faecalis ATCC 6055	EOK09817.1
Enterococcus faecalis B373	ETU56355.1
Enterococcus faecalis B375	ETU59690.1
Enterococcus faecalis B388	ETU66734.1
Enterococcus faecalis BM4654	ERT22326.1
Enterococcus faecalis CH188	EEU83936.1
Enterococcus faecalis DS5	EEU65361.1
Enterococcus faecalis EnGen0067	EOE36355.1
Enterococcus faecalis EnGen0068	EOK60899.1
Enterococcus faecalis EnGen0069	EOK61941.1
Enterococcus faecalis EnGen0070	EOE40348.1
Enterococcus faecalis EnGen0078	EOE07317.1
Enterococcus faecalis EnGen0079	EOE11069.1
Enterococcus faecalis EnGen0085	EOE69387.1
Enterococcus faecalis EnGen0087	EOF17228.1
Enterococcus faecalis EnGen0088	EOE45978.1
Enterococcus faecalis EnGen0089	EOE52128.1
Enterococcus faecalis EnGen0090	EOE54055.1
Enterococcus faecalis EnGen0091	EOE59226.1
Enterococcus faecalis EnGen0092	EOE68244.1
Enterococcus faecalis EnGen0093	EOE77632.1
Enterococcus faecalis EnGen0094	EOE81271.1
Enterococcus faecalis EnGen0095	EOE86625.1
Enterococcus faecalis EnGen0096	EOE88375.1
Enterococcus faecalis EnGen0097	EOE93276.1
Enterococcus faecalis EnGen0098	EOF02369.1
Enterococcus faecalis EnGen0099	EOF03324.1
Enterococcus faecalis EnGen0100	EOF12774.1
Enterococcus faecalis EnGen0101	EOF34985.1
Enterococcus faecalis EnGen0106	EOE37509.1
Enterococcus faecalis EnGen0107	EOF15906.1
Enterococcus faecalis EnGen0108	EOF22854.1
Enterococcus faecalis EnGen0109	EOE67233.1
Enterococcus faecalis EnGen0110	EOE60833.1
Enterococcus faecalis EnGen0111	EOE71939.1
Enterococcus faecalis EnGen0112	EOE98929.1
Enterococcus faecalis EnGen0113	EOF06702.1
Enterococcus faecalis EnGen0114	EOF06780.1
Enterococcus faecalis EnGen0119	EOE78791.1
Enterococcus faecalis EnGen0120	EOE50960.1
Enterococcus faecalis EnGen0194	EOG32052.1
Enterococcus faecalis EnGen0195	EOG41201.1
Enterococcus faecalis EnGen0196	EOG40520.1
Enterococcus faecalis EnGen0197	EOG45747.1
Enterococcus faecalis EnGen0198	EOG49159.1
Enterococcus faecalis EnGen0199	EOG52184.1
Enterococcus faecalis EnGen0200	EOG55956.1
Enterococcus faecalis EnGen0201	EOM47171.1
Enterococcus faecalis EnGen0202	EOM47547.1
Enterococcus faecalis EnGen0203	EOM54058.1
Enterococcus faecalis EnGen0204	EOG59260.1
Enterococcus faecalis EnGen0205	EOG64505.1
Enterococcus faecalis EnGen0206	EOG71661.1
Enterococcus faecalis EnGen0207	EOG59433.1
Enterococcus faecalis EnGen0208	EOG77170.1
Enterococcus faecalis EnGen0209	EOG81470.1
Enterococcus faecalis EnGen0210	EOG85030.1
Enterococcus faecalis EnGen0211	EOG83552.1
Enterococcus faecalis EnGen0212	EOG90137.1
Enterococcus faecalis EnGen0213	EOG93561.1
Enterococcus faecalis EnGen0214	EOG94599.1
Enterococcus faecalis EnGen0215	EOG99089.1
Enterococcus faecalis EnGen0216	EOH04293.1
Enterococcus faecalis EnGen0217	EOH03814.1
Enterococcus faecalis EnGen0218	EOH06555.1
Enterococcus faecalis EnGen0219	EOH14023.1
Enterococcus faecalis EnGen0220	EOH14498.1
Enterococcus faecalis EnGen0221	EOH15060.1
Enterococcus faecalis EnGen0222	EOH24906.1
Enterococcus faecalis EnGen0223	EOH23478.1
Enterococcus faecalis EnGen0224	EOH25096.1
Enterococcus faecalis EnGen0225	EOH33630.1
Enterococcus faecalis EnGen0226	EOH33308.1
Enterococcus faecalis EnGen0228	EOG68742.1
Enterococcus faecalis EnGen0231	EOI26955.1
Enterococcus faecalis EnGen0232	EOM28329.1
Enterococcus faecalis EnGen0235	EOH60041.1
Enterococcus faecalis EnGen0244	EOI09977.1
Enterococcus faecalis EnGen0245	EOL60151.1
Enterococcus faecalis EnGen0246	EOL64825.1
Enterococcus faecalis EnGen0247	EOL68252.1
Enterococcus faecalis EnGen0251	EOI27763.1
Enterococcus faecalis EnGen0252	EOI19958.1
Enterococcus faecalis EnGen0253	EOM22453.1
Enterococcus faecalis EnGen0280	EOL73649.1
Enterococcus faecalis EnGen0298	EOI96701.1
Enterococcus faecalis EnGen0302	EOJ00228.1
Enterococcus faecalis EnGen0303	EOL87177.1
Enterococcus faecalis EnGen0306	EOJ07468.1
Enterococcus faecalis EnGen0310	EOI79100.1
Enterococcus faecalis EnGen0311	EOI95446.1
Enterococcus faecalis EnGen0334	EOK27181.1
Enterococcus faecalis EnGen0336	EOJ65123.1
Enterococcus faecalis EnGen0337	EOJ74371.1
Enterococcus faecalis EnGen0338	EOK19004.1
Enterococcus faecalis EnGen0340	EOJ96539.1
Enterococcus faecalis EnGen0344	EOL96003.1
Enterococcus faecalis EnGen0348	EOL36132.1
Enterococcus faecalis EnGen0355	EOJ75184.1
Enterococcus faecalis EnGen0359	EOJ95323.1
Enterococcus faecalis EnGen0360	EOK03757.1
Enterococcus faecalis EnGen0362	EOL34134.1
Enterococcus faecalis EnGen0374	EOG72352.1
Enterococcus faecalis GA2	KAJ57765.1
Enterococcus faecalis GAN13	KAJ80272.1
Enterococcus faecalis MD6	KAJ71606.1
Enterococcus faecalis MN16	KAJ64407.1
Enterococcus faecalis MTUP9	KAJ81196.1
Enterococcus faecalis MTmid8	KAJ70704.1
Enterococcus faecalis NJ44	KAJ85908.1
Enterococcus faecalis TX0027	EFT47976.1
Enterococcus faecalis TX0309A	EFU95029.1
Enterococcus faecalis TX0470	EFQ69779.1
Enterococcus faecalis TX0645	EFU04723.1
Enterococcus faecalis TX1341	EFU13276.1
Enterococcus faecalis TX4244	EFT92251.1
Enterococcus faecalis TX4248	EFM84307.1
Enterococcus flavescens ATCC 49996	EOH83464.1
Exiguobacterium mexicanum	KGI86370.1
Lactobacillus crispatus	WP_005721911.1
Lactobacillus crispatus 125-2-CHN	EEU18905.1
Lactobacillus crispatus 2029	ETW72903.1
Lactobacillus crispatus CTV-05	EFQ45481.1
Lactobacillus crispatus FB049-03	EKB60155.1
Lactobacillus crispatus FB077-07	EKB62797.1
Lactobacillus crispatus MV-1A-US	EEU28751.1
Lactobacillus crispatus MV-3A-US	EEX30204.1
Lactobacillus helveticus	WP_014918795.1
Lactobacillus helveticus R0052	YP_006655842.1
Lactococcus lactis	WP_029344607.1
Listeria innocua	AHK25022.1
Listeria innocua ATCC 33091	EHN61024.1
Listeria monocytogenes	KEX21484.1
Listeria monocytogenes 07PF0776	AFH79679.1
Listeria monocytogenes FSL N1-017	EFK41963.1
Listeria monocytogenes FSL R2-503	EEW18969.1
Listeria monocytogenes J1816	EGF37831.1
Listeria monocytogenes L312	YP_006754227.1
Listeria monocytogenes SLCC2378	CBY72882.1
Listeria monocytogenes SLCC2755	CBY48694.1
Listeria monocytogenes WSLC1042	AHJ35174.1
Listeria monocytogenes serotype 4b str. CLIP 80459	YP_002757821.1
Listeria monocytogenes serotype 4b str. F2365	AAT03895.1
Listeria monocytogenes serotype 4b str. LL195	CCO63704.1
Listeria monocytogenes serotype 4bV str. LS542	ERH87138.1
Listeria monocytogenes serotype 4bV str. LS642	ERH79260.1
Listeria monocytogenes serotype 4bV str. LS643	ERH87937.1
Listeria monocytogenes serotype 4bV str. LS644	ERH82705.1
Listeria monocytogenes serotype 4bV str. LS645	ERH81297.1
Listeria monocytogenes serotype 7 str. SLCC2482	YP_006693101.1
Listeria monocytogenes str. 4b H7858	EAL10365.1
Listeria seeligeri FSL S4-171	EFS02713.1
Mobiluncus mulieris	WP_004018421.1
Mobiluncus mulieris FB024-16	EFN92445.1
Oenococcus kitaharae	WP_007745654.1
Oenococcus kitaharae DSM 17330	EHN58992.1
Propionibacterium	WP_002519916.1
Propionibacterium acnes	WP_013069418.1
Propionibacterium acnes HL005PA1	EFT29310.1
Propionibacterium acnes HL007PA1	EFS69869.1
Propionibacterium acnes HL038PA1	EFT69160.1
Propionibacterium acnes HL043PA1	EGE95685.1
Propionibacterium acnes HL043PA2	EGE93488.1
Propionibacterium acnes HL045PA1	EFT21539.1
Propionibacterium acnes HL053PA1	EFT19487.1
Propionibacterium acnes HL056PA1	EFS70668.1
Propionibacterium acnes HL074PA1	EFS37246.1
Propionibacterium acnes HL078PA1	EFT54407.1
Propionibacterium acnes HL083PA1	EFS49402.1
Propionibacterium acnes HL086PA1	EFS77213.1
Propionibacterium acnes HL096PA1	YP_007886541.1
Propionibacterium acnes HL096PA2	EGE74424.1
Propionibacterium acnes HL099PA1	EGF73205.1
Propionibacterium acnes SK137	ADD99586.1
Propionibacterium acnes SK182	EGR94682.1
Propionibacterium sp. KPL1854	ERS36581.1
Propionibacterium sp. KPL2009	ERS25567.1
Staphylococcus aureus	WP_000627618.1
Staphylococcus aureus RF122	YP_416846.1
Staphylococcus aureus subsp. *aureus* JKD6159	ADL23375.1
Staphylococcus aureus subsp. *aureus* USA300_TCH959	EES92840.1
Staphylococcus lugdunensis	WP_002492067.1
Staphylococcus lugdunensis ACS-027-V-Sch2	EKS22906.1
Staphylococcus lugdunensis HKU09-01	YP_003470294.1
Staphylococcus lugdunensis M23590	EFU83565.1
Staphylococcus lugdunensis N920143	YP_005759171.1
Staphylococcus lugdunensis UCIM6116	EVI52859.1
Staphylococcus lugdunensis VCU139	EHS05291.1
Staphylococcus lugdunensis VCU148	KAK62221.1
Staphylococcus lugdunensis VCU150	KAK58134.1
Streptococcus anginosus	WP_003036343.1
Streptococcus anginosus C238	YP_008500116.1
Streptococcus anginosus SK52 = DSM 20563	GAD36919.1
Streptococcus anginosus T5	GAD46826.1
Streptococcus anginosus group	WP_003031070.1
Streptococcus anginosus subsp. *whileyi*	BAO73279.1
Streptococcus anginosus subsp. *whileyi* CCUG 39159	EID21406.1
Streptococcus anginosus subsp. *whileyi* MAS624	BAN62320.1
Streptococcus canis	WP_003047821.1
Streptococcus canis FSL Z3-227	EIQ81603.1
Streptococcus castoreus	WP_027969506.1
Streptococcus constellatus	WP_006267405.1
Streptococcus constellatus subsp. *constellatus*	BAO73259.1
Streptococcus constellatus subsp. *pharyngis*	BAO73270.1
Streptococcus constellatus subsp. *pharyngis* C1050	AGU79357.1
Streptococcus constellatus subsp. *pharyngis* C232	AGU72233.1
Streptococcus constellatus subsp. *pharyngis* C818	AGU73989.1
Streptococcus constellatus subsp. *pharyngis* SK1060 = CCUG 46377	EGV10767.1
Streptococcus constellatus subsp. *viborgensis* SK1359	BAO73288.1
Streptococcus didelphis	WP_018366953.1
Streptococcus dysgalactiae	WP_015016859.1
Streptococcus dysgalactiae subsp. *equisimilis* 167	BAN93159.1
Streptococcus dysgalactiae subsp. *equisimilis* AC-2713	YP_006904304.1
Streptococcus dysgalactiae subsp. *equisimilis* ATCC 12394	YP_006012853.1
Streptococcus dysgalactiae subsp. *equisimilis* GGS_124	BAH81209.1
Streptococcus dysgalactiae subsp. *equisimilis* RE378	YP_006089332.1
Streptococcus dysgalactiae subsp. *equisimilis* SK1249	EGL48335.1
Streptococcus dysgalactiae subsp. *equisimilis* SK1250	EGR89087.1
Streptococcus equi	WP_021320450.1
Streptococcus equi subsp. *equi* 4047	CAW92802.1
Streptococcus equi subsp. *ruminatorum* CECT 5772	KED03797.1
Streptococcus equi subsp. *zooepidemicus*	CAX00154.1
Streptococcus equi subsp. *zooepidemicus* ATCC 35246	AEJ24716.1
Streptococcus equi subsp. *zooepidemicus* CY	AIA67973.1
Streptococcus equi subsp. *zooepidemicus* MGCS10565	YP_002122878.1
Streptococcus equi subsp. *zooepidemicus* SzS31A1	EQB24140.1
Streptococcus ictaluri	WP_008087931.1
Streptococcus ictaluri 707-05	EHI70399.1
Streptococcus iniae	AAN16974.1
Streptococcus iniae 9117	EKB52950.1
Streptococcus iniae IUSA1	ESR10088.1
Streptococcus iniae SF1	YP_008056991.1
Streptococcus intermedius SK54	GAD40641.1
Streptococcus phocae C-4	KGR72224.1
Streptococcus porcinus	WP_003085810.1
Streptococcus porcinus str. Jelinkova 176	EGJ28166.1
Streptococcus pseudoporcinus	WP_007895544.1
Streptococcus pseudoporcinus LQ 940-04	EHI65561.1
Streptococcus pseudoporcinus SPIN 20026	EFR43668.1
Streptococcus pyogenes	AIL11847.1
Streptococcus pyogenes A20	YP_006932647.1
Streptococcus pyogenes AA216	KGE56761.1
Streptococcus pyogenes AA472	KGE55663.1
Streptococcus pyogenes ABC020004984	EZL07464.1
Streptococcus pyogenes ABC020005405	EZL04521.1
Streptococcus pyogenes ABC020005716	EZM86425.1
Streptococcus pyogenes ABC020005773	EZM98094.1
Streptococcus pyogenes ABC020005887	EZL66568.1
Streptococcus pyogenes ABC020006103	EZL69740.1
Streptococcus pyogenes ABC020006298	EZL01609.1
Streptococcus pyogenes ABC020006309	EZK99877.1
Streptococcus pyogenes ABC020006345	EZM90158.1
Streptococcus pyogenes ABC020006715	EZM91974.1
Streptococcus pyogenes ABC020007794	EZL00284.1
Streptococcus pyogenes ABC020009156	EZK97848.1
Streptococcus pyogenes ABC020010041	EZK94809.1
Streptococcus pyogenes ABC020013256	EZN18369.1
Streptococcus pyogenes ABC020013551	EZK93305.1
Streptococcus pyogenes ABC020013952	EZL68340.1
Streptococcus pyogenes ABC020014529	EZL63105.1
Streptococcus pyogenes ABC020014690	EZK92279.1
Streptococcus pyogenes ABC020014764	EZM94430.1
Streptococcus pyogenes ABC020014897	EZL62452.1
Streptococcus pyogenes ABC020014925	EZL60662.1
Streptococcus pyogenes ABC020015277	EZK89616.1
Streptococcus pyogenes ABC020015285	EZK91630.1
Streptococcus pyogenes ABC020015292	EZK83714.1
Streptococcus pyogenes ABC020015294	EZK84743.1
Streptococcus pyogenes ABC020016937	EZK86148.1
Streptococcus pyogenes ABC020017280	EZN20443.1
Streptococcus pyogenes ABC020017526	EZL59288.1
Streptococcus pyogenes ABC020017774	EZN21628.1
Streptococcus pyogenes ABC020021452	EZN23373.1
Streptococcus pyogenes ABC020025676	EZK78818.1
Streptococcus pyogenes ABC020026287	EZL54648.1
Streptococcus pyogenes ABC020026799	EZL57966.1
Streptococcus pyogenes ABC020026946	EZK80982.1
Streptococcus pyogenes ABC020029706	EZM22867.1
Streptococcus pyogenes ABC020029711	EZL53727.1
Streptococcus pyogenes ABC020029793	EZL50852.1
Streptococcus pyogenes ABC020030020	EZM96178.1
Streptococcus pyogenes ABC020030063	EZL95891.1
Streptococcus pyogenes ABC020030925	EZL49398.1
Streptococcus pyogenes ABC020031290	EZL48255.1
Streptococcus pyogenes ABC020031898	EZK78048.1
Streptococcus pyogenes ABC020032057	EZN01129.1
Streptococcus pyogenes ABC020032182	EZL46699.1
Streptococcus pyogenes ABC020032183	EZK76765.1
Streptococcus pyogenes ABC020032186	EZM21945.1
Streptococcus pyogenes ABC020032523	EZL44883.1
Streptococcus pyogenes ABC020032535	EZM99319.1
Streptococcus pyogenes ABC020033020	EZL94073.1
Streptococcus pyogenes ABC020033193	EZL43961.1
Streptococcus pyogenes ABC020033233	EZM25036.1
Streptococcus pyogenes ABC020033799	EZL40926.1
Streptococcus pyogenes ABC020033919	EZM19899.1
Streptococcus pyogenes ABC020035427	EZK76235.1
Streptococcus pyogenes ABC020035446	EZM17426.1
Streptococcus pyogenes ABC020037542	EZM16213.1
Streptococcus pyogenes ABC020038544	EZM48551.1
Streptococcus pyogenes ABC020038545	EZN02450.1
Streptococcus pyogenes ABC020038558	EZL37853.1
Streptococcus pyogenes ABC020039031	EZM15684.1
Streptococcus pyogenes ABC020039391	EZL38778.1
Streptococcus pyogenes ABC020040643	EZM43240.1
Streptococcus pyogenes ABC020041397	EZN25885.1
Streptococcus pyogenes ABC020041424	EZM10046.1
Streptococcus pyogenes ABC020043540	EZL91280.1
Streptococcus pyogenes ABC020044010	EZK71661.1
Streptococcus pyogenes ABC020044173	EZL35755.1
Streptococcus pyogenes ABC020044188	EZL34919.1
Streptococcus pyogenes ABC020044193	EZL33973.1
Streptococcus pyogenes ABC020044203	EZL84609.1
Streptococcus pyogenes ABC020044412	EZK69905.1
Streptococcus pyogenes ABC020046158	EZM40179.1
Streptococcus pyogenes ABC020046230	EZM59851.1
Streptococcus pyogenes ABC020046264	EZL31600.1
Streptococcus pyogenes ABC020046470	EZL25746.1
Streptococcus pyogenes ABC020046534	EZM34639.1
Streptococcus pyogenes ABC020046559	EZM38035.1
Streptococcus pyogenes ABC020046589	EZN29605.1
Streptococcus pyogenes ABC020046688	EZN27624.1
Streptococcus pyogenes ABC020046986	EZN30941.1
Streptococcus pyogenes ABC020047076	EZL79721.1
Streptococcus pyogenes ABC020047086	EZM60893.1
Streptococcus pyogenes ABC020047328	EZN32978.1
Streptococcus pyogenes ABC020047395	EZK70416.1
Streptococcus pyogenes ABC020047506	EZM54206.1
Streptococcus pyogenes ABC020047619	EZL74515.1
Streptococcus pyogenes ABC020047635	EZM55483.1
Streptococcus pyogenes ABC020047925	EZK65930.1
Streptococcus pyogenes ABC020047955	EZM61725.1
Streptococcus pyogenes ABC020047959	EZK65117.1
Streptococcus pyogenes ABC020047977	EZM76338.1
Streptococcus pyogenes ABC020047993	EZL27947.1
Streptococcus pyogenes ABC020048184	EZK63120.1
Streptococcus pyogenes ABC020048262	EZM34042.1
Streptococcus pyogenes ABC020048387	EZM79356.1
Streptococcus pyogenes ABC020048395	EZL28801.1
Streptococcus pyogenes ABC020048503	EZM63476.1
Streptococcus pyogenes ABC020048541	EZK62834.1
Streptococcus pyogenes ABC020048543	EZL23177.1
Streptococcus pyogenes ABC020048606	EZN06458.1
Streptococcus pyogenes ABC020048885	EZM04805.1
Streptococcus pyogenes ABC020049250	EZL20252.1
Streptococcus pyogenes ABC020049540	EZM02720.1
Streptococcus pyogenes ABC020049545	EZM65212.1
Streptococcus pyogenes ABC020050193	EZN33996.1
Streptococcus pyogenes ABC020050731	EZL21730.1
Streptococcus pyogenes ABC020050818	EZN04456.1
Streptococcus pyogenes ABC020051161	EZM08650.1
Streptococcus pyogenes ABC020051269	EZN39726.1
Streptococcus pyogenes ABC020052023	EZN36612.1
Streptococcus pyogenes ABC020052203	EZL18913.1
Streptococcus pyogenes ABC020052211	EZM58730.1
Streptococcus pyogenes ABC020052216	EZL17908.1
Streptococcus pyogenes ABC020052218	EZN07637.1
Streptococcus pyogenes ABC020052291	EZK60904.1
Streptococcus pyogenes ABC020052309	EZM66994.1
Streptococcus pyogenes ABC020052313	EZM68739.1
Streptococcus pyogenes ABC020052375	EZN09671.1
Streptococcus pyogenes ABC020052378	EZL90141.1
Streptococcus pyogenes ABC020052420	EZL15911.1
Streptococcus pyogenes ABC020052497	EZL75544.1
Streptococcus pyogenes ABC020052519	EZM03953.1
Streptococcus pyogenes ABC020052543	EZN40534.1
Streptococcus pyogenes ABC020052553	EZN11415.1
Streptococcus pyogenes ABC020052558	EZK56964.1
Streptococcus pyogenes ABC020052877	EZN13714.1
Streptococcus pyogenes ABC020052898	EZM77592.1
Streptococcus pyogenes ABC020052980	EZM70484.1
Streptococcus pyogenes ABC020053060	EZM45746.1
Streptococcus pyogenes ABC020053240	EZM01429.1
Streptococcus pyogenes ABC020053250	EZM84630.1
Streptococcus pyogenes ABC020054184	EZM44515.1
Streptococcus pyogenes ABC020054871	EZL13291.1
Streptococcus pyogenes ABC020054955	EZL12666.1
Streptococcus pyogenes ABC020054973	EZK55532.1
Streptococcus pyogenes ABC020055897	EZM00332.1
Streptococcus pyogenes ABC020056020	EZM12753.1
Streptococcus pyogenes ABC020056060	EZL83926.1
Streptococcus pyogenes ABC020056064	EZL97559.1
Streptococcus pyogenes ABC020056068	EZM82887.1
Streptococcus pyogenes ABC020056181	EZN15172.1
Streptococcus pyogenes ABC020056765	EZM72615.1
Streptococcus pyogenes ABC020056794	EZM33516.1
Streptococcus pyogenes ABC020056883	EZN17038.1
Streptococcus pyogenes ABC020056884	EZM56071.1
Streptococcus pyogenes ABC020056885	EZL09064.1
Streptococcus pyogenes ABC020056891	EZL78578.1
Streptococcus pyogenes ABC020056894	EZM50647.1
Streptococcus pyogenes ABC020056898	EZL76407.1
Streptococcus pyogenes ABC020057168	EZL91515.1
Streptococcus pyogenes ABC020057192	EZL70617.1
Streptococcus pyogenes ABC020057288	EZM10802.1
Streptococcus pyogenes ABC020057442	EZM74377.1
Streptococcus pyogenes ABC020057449	EZL85075.1
Streptococcus pyogenes ABC020058759	EZL05836.1
Streptococcus pyogenes ABC020059502	EZM81136.1
Streptococcus pyogenes ABC020060220	EZM27129.1
Streptococcus pyogenes ABC020060777	EZM49091.1
Streptococcus pyogenes ABC020060793	EZN37311.1
Streptococcus pyogenes ABC020061424	EZM88171.1
Streptococcus pyogenes ABC020062474	EZM28932.1
Streptococcus pyogenes ABC020062601	EZM29499.1
Streptococcus pyogenes ABC020064181	EZM38970.1
Streptococcus pyogenes ATCC 10782	EFM33242.1
Streptococcus pyogenes Alab49	YP_006071750.1
Streptococcus pyogenes GA03455	ESU92116.1
Streptococcus pyogenes GA03747	ESU92489.1
Streptococcus pyogenes GA03799	ESU85817.1
Streptococcus pyogenes GA03805	ESA58106.1
Streptococcus pyogenes GA06023	ERL14940.1
Streptococcus pyogenes GA16797	ESU93624.1
Streptococcus pyogenes GA19681	EQL81989.1
Streptococcus pyogenes GA19700	ESA44718.1
Streptococcus pyogenes GA19702	ESU91867.1
Streptococcus pyogenes GA40056	ESA53006.1
Streptococcus pyogenes GA40377	ESA58487.1
Streptococcus pyogenes GA40468	ESA52401.1
Streptococcus pyogenes GA40634	EPZ47781.1
Streptococcus pyogenes GA40884	ESU88551.1
Streptococcus pyogenes GA41039	ESA46931.1
Streptococcus pyogenes GA41046	ERL18782.1
Streptococcus pyogenes GA41208	ESA50455.1
Streptococcus pyogenes GA41345	EPZ46951.1
Streptococcus pyogenes GA41394	ESA54459.1
Streptococcus pyogenes HKU QMH11M0907901	EIK42294.1
Streptococcus pyogenes HSC5	AGQ27390.1
Streptococcus pyogenes M1 476	YP_007587151.1
Streptococcus pyogenes M1 GAS	NP_268966.1
Streptococcus pyogenes MGAS10270	ABF33684.1
Streptococcus pyogenes MGAS10394	YP_059899.1
Streptococcus pyogenes MGAS10750	ABF37597.1
Streptococcus pyogenes MGAS15252	YP_005388641.1
Streptococcus pyogenes MGAS1882	YP_005411340.1
Streptococcus pyogenes MGAS2096	YP_600222.1
Streptococcus pyogenes MGAS2111	KGE60575.1
Streptococcus pyogenes MGAS315	AAM79090.1
Streptococcus pyogenes MGAS5005	AAZ51183.1
Streptococcus pyogenes MGAS6180	AAX71657.1
Streptococcus pyogenes MGAS8232	NP_606967.1
Streptococcus pyogenes MGAS9429	ABF31805.1
Streptococcus pyogenes NS88.2	CCG27133.1
Streptococcus pyogenes NZ131	YP_002285592.1
Streptococcus pyogenes SS1447	KGE59378.1
Streptococcus pyogenes SSI-1	BAC64466.1
Streptococcus pyogenes STAB1102	AIG48613.1
Streptococcus pyogenes STAB901	AIG50128.1
Streptococcus pyogenes STAB902	AIG47640.1
Streptococcus pyogenes UTMEM-1	EQL80632.1
Streptococcus pyogenes UTSW-2	EQL82124.1
Streptococcus pyogenes str. Manfredo	YP_001128783.1
Virgibacillus alimentarius	WP_029270583.1
